# Micropillars in Cell Mechanobiology: Design, Fabrication, Characterization, and Biosensing Applications

**DOI:** 10.1002/smsc.202400410

**Published:** 2024-12-09

**Authors:** Prabuddha De Saram, Nam‐Trung Nguyen, Sina Jamali, Navid Kashaninejad

**Affiliations:** ^1^ Queensland Micro‐ and Nanotechnology Centre Nathan Campus Griffith University 170 Kessels Road Brisbane QLD 4111 Australia

**Keywords:** biosensings, mechanobiologies, mechano‐stimulations, micropillars

## Abstract

Eukaryotic cells possess the remarkable ability to sense and respond to mechanical cues from their extracellular environment, a phenomenon known as mechanobiology, which is crucial for the proper functioning of biological systems. Micropillars have emerged as a prominent tool for quantifying cellular forces and have demonstrated versatility beyond force measurement, including the modulation of the extracellular environment and the facilitation of mechano‐stimulation. In this comprehensive review, innovative strategies in micropillars’ design, fabrication, characterization, and biosensing applications are explored. The review begins with a foundational overview of micropillar‐based cell mechanobiology studies to provide a complete understanding, and then it delves into novel methodologies within each domain. The latter part of the review unveils innovative micropillars’ applications beyond mechanobiology, such as their use in enhancing biosensing surfaces and as upstream fluid manipulators for biosensors. Finally, in this review, future research directions are discussed and the current limitations of these techniques are outlined. Despite the extensive exploration of micropillar applications, a significant gap remains between research advancements and the practical implementation of micropillars in point‐of‐care diagnostics. Bridging this gap is crucial for translating laboratory innovations into real‐world medical and diagnostic tools.

## Graphical abstract

In this article, the pivotal role of micropillars in cell mechanobiology is explored, detailing their design, fabrication, and characterization. Novel applications in biosensing are highlighted, emphasizing their ability to modulate cellular environments and facilitate mechano‐stimulation. Future research directions and practical limitations are discussed, offering insights into the potential of micropillars in diagnostics and biosensing technologies.

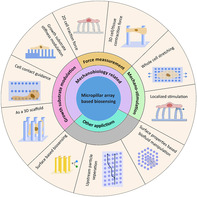

## 1 Introduction

Both chemical and physical surface modifications are widely employed strategies to regulate cell responses and their interactions with material surfaces.^[^ ^1^, ^2^, ^3^ ^]^ Among these, topographical modifications, such as surface roughness, porosity, and micro/nanoscale patterning, have found extensive applications in cell research, tissue engineering, biosensor design, and the development of advanced medical devices.^[^ ^4^, ^5^, ^6^ ^]^ Micropillar arrays, owing to their quasi‐3D topography, stand out among surface patterning techniques.^[^ ^7^ ^]^ Over the past two decades, there has been a growing interest in micropillar‐based biosensing. These arrays play a crucial role in enhancing established sensing principles and enabling the development of novel sensing techniques. The precise control and superior geometrical properties of micropillars significantly improve the performance of surface‐based sensing technologies, particularly in bio‐interfaces. Notably, their distinct role in mechanobiology‐related studies stands out among the various applications of micropillar arrays.

Mechanical forces between the extracellular matrix (ECM) and cells regulate various cellular functions, including cellular adhesion, migration, differentiation, proliferation, and morphogenesis.^[^ ^8^ ^]^ Such forces arise from external influences on the ECM or intracellular actin–myosin contractions.^[^ ^9^ ^]^ The transfer of cell‐generated forces between the ECM and the intracellular actin cytoskeleton occurs through intercellular protein complexes known as focal adhesions, and these forces are termed cell traction forces (CTFs).^[^ ^10^ ^]^ Estimating CTFs provides valuable insights into cellular mechanotransduction and its associated biological phenomena, such as wound healing, embryogenesis, immune response, and cancer metastasis.^[^ ^11^ ^]^

In 1980, Harris et al. introduced the utilization of wrinkles formed by a cell on continuous silicon/gel substrates to estimate CTFs.^[^ ^12^ ^]^ Subsequently, Dembo and Wang embedded fluorescent beads on the gel surface to precisely quantify surface displacements.^[^ ^13^ ^]^ Traction force microscopy (TFM) employs this principle for CTF measurements. However, the mechanical coupling of beads, the complexity inherent in force calculations, and the challenges associated with accurately identifying focal adhesion locations present significant limitations to this method.^[^ ^14^ ^]^

The concept of using micropillar arrays for CTF measurement was first proposed by Tan et al. in 2003. Subsequently, micropillar arrays have gained popularity as a technique for measuring CTFs.^[^ ^15^ ^]^ In micropillar arrays, pillar deflection directly measures the local force on each pillar independently of the forces acting on neighboring pillars, eliminating the aforementioned limitations of continuous substrates. However, the natural ECM is a continuous environment, unlike the discrete nature of micropillar arrays, and continuous gel substrates offer a more accurate mimicry of the natural topology of the ECM.^[^ ^16^, ^17^, ^18^, ^19^ ^]^

In addition to generating forces, focal adhesions sense mechanical cues from the ECM and transduce them into biochemical signals, a process known as mechanotransduction.^[^ ^20^ ^]^ This feedback mechanism influences CTFs and ECM gene expression. Before the advent of micropillars, Dembo and Wang explored continuous polyacrylamide substrates with tunable mechanical properties to study stiffness‐driven cell mechanotransduction.^[^ ^13^ ^]^ However, micropillars provide extensive controllability over ECM mechanical properties, including micro/nanorigidity, adhesion topology, and anisotropic properties. This versatility positions micropillars as a valuable tool for ECM studies. Furthermore, integrating active actuation techniques with micropillars facilitates CTF studies during mechano‐stimulations.

Several high‐quality reviews have been published on micropillar‐based mechanobiology studies. Digabel et al. and Obenaus et al. extensively reviewed the use of deformable structures for CTF measurement, offering comparative analyses of micropillar arrays and TFM.^[^ ^21^, ^22^ ^]^ Polacheck et al. also reviewed different available methods of CTF measurement. They have extended their review to independent substrate deformation methods, such as molecular force sensors.^[^ ^23^ ^]^ Sniadecki et al. and Gupta et al. have detailed the process of fabricating polydimethylsiloxane (PDMS) micropillars for CTF measurement,^[^ ^7^, ^24^ ^]^ and Ribeiro et al. provided a comprehensive discussion of the design parameters of pillar arrays and their impact on the performance of sensors.^[^ ^25^ ^]^ In their review, Xu et al. focused on the diverse applications of micropillars in mechanobiology, particularly emphasizing CTFs and discussing ECM modulation.^[^ ^26^ ^]^ Long et al. reviewed the applications of micropillars in biomechanics, mainly focusing on stem cell differentiation.^[^ ^27^ ^]^ Park et al. discussed the fabrication and application of different stimuli‐responsive micropillar arrays, which show significant potential for biological applications.^[^ ^28^ ^]^

**Figure 1:**
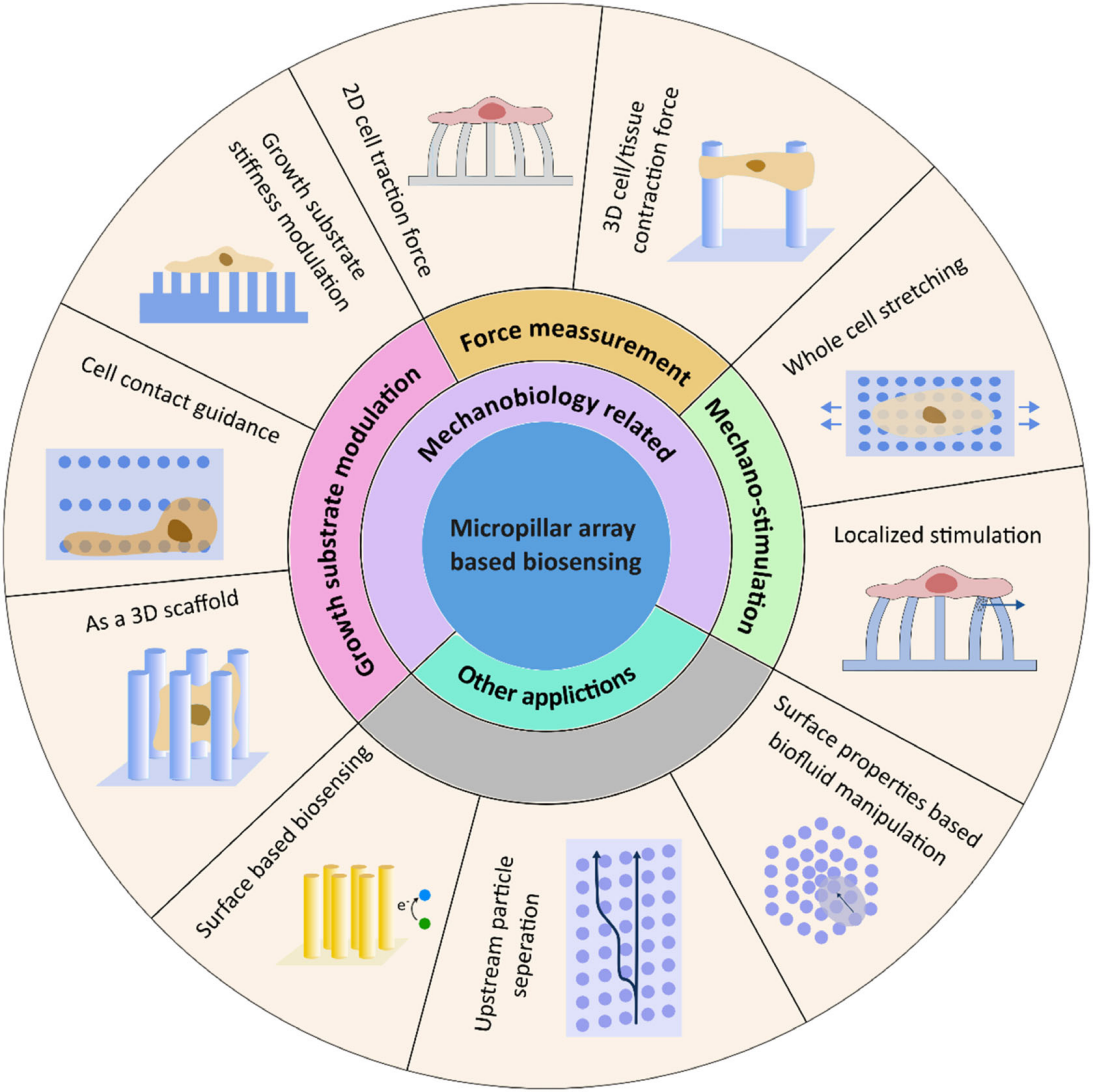
The overall scope of the present study. The focus is concentrated on cell‐mechanobiology‐related applications of micropillar arrays. This area can be divided into three parts: growth substrate modulation, force measurement, and mechano‐stimulation. Apart from mechanobiology, micropillars can be used as electrodes in biosensors, bioparticle separation, and biofluid manipulation.

While previous reviews have primarily focused on established methodologies and applications of micropillars in cell mechanobiology, our present review explores emerging trends and areas that have not received significant attention. Specifically, we provide in‐depth coverage of emerging fabrication methods, advanced 3D force measurement techniques, and innovative biosensing applications of micropillars. **Figure** **1** summarizes the overall scope of the current review. The initial section covers the design and fabrication of micropillars. In addition to conventional techniques, we explore other manufacturing methods that are not commonly utilized but show potential. In the third, fourth, and fifth sections, we discuss cell‐mechanobiology‐related applications of micropillars, where we review innovative approaches, such as 3D force measurements and 3D ECM mimicking, alongside standard techniques. In the final section, we examine non‐mechanobiology‐related biosensing applications of micropillars, such as surface‐property‐based biosensing and fluid manipulation.

## 2 Micropillar Design and Fabrication

**Figure 2:**
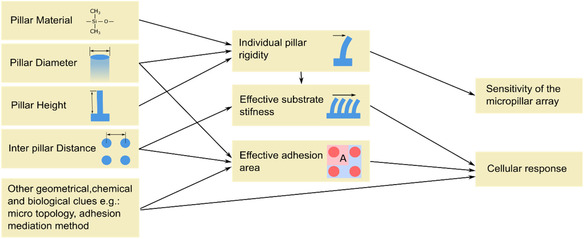
The influence of material and geometrical parameters on the performance of micropillar‐based cell mechanobiology studies.

The design of micropillars heavily relies on the intended application and the expected sensitivity. Micropillar material, pillar diameter, height, and interpillar distance (ID) are essential parameters that define the micropillar array sensitivity and cellular response, see **Figure** **2**. Additional parameters, such as individual pillar topology, nanoscale surface modification, and pillar arrangement geometry, mediate specific substrate properties.

In cellular force measurement, selected materials should be biocompatible and must be able to adhere to cells directly or through external protein absorption. Generally, polymers are suitable candidates for mechanobiology studies due to the compatibility of their stiffness with biological cells and the ability to modify the stiffness in the polymerization step. This allows polymers to have pillars with larger diameters than metals such as silicon, which is widely used in microfabrication. Particularly in measuring adhesion‐mediated CTFs, the pillar diameter should ensure sufficient top area for focal adhesions.^[^ ^29^, ^30^ ^]^ Alternative methods, such as wider top attachment pads, can be used for stiffer materials requiring smaller diameters for rigidity regulation. ID affects the continuity of the extracellular environment experienced by the cells. Smaller IDs more closely mimic the continuous ECM but geometrically limit the pillar displacement. Conversely, larger pillar gaps may lead to cells driving into interpillar gaps, cellular contact with the bottom surface, and non‐tangential forces on pillar tops.^[^ ^31^ ^]^ Most CTF studies have kept pillar diameter within 1–5 μm (for PDMS) and center‐to‐center pillar distance close to twice the pillar diameter.

In addition to the aforementioned considerations, pillar stability warrants attention, particularly for high aspect ratio micropillars, which are prone to collapse under fluid surface tension forces during fabrication and experiments. The maintenance of micropillar stability under fluid surface tension is discussed elsewhere.^[^ ^32^ ^]^ Moreover, if microcontact printing of proteins or dyes on pillars is necessary, appropriate support structures must be designed to prevent pillar collapse upon contact.^[^ ^15^ ^]^ **Table** **1** summarizes the micropillar design parameters used in the literature.

**Table 1:** Micropillar design parameters, fabrication methods, and visualization techniques in mechanobiology experiments.

Pillar diameter [μm]	Height [μm]	Inter pillar distance [μm]	Pillar arrangement	Material	Fabrication methoda)	Cell type	Application	Functionalization/passivation	Displacement measurement (visualization and dye/enhancement)	References
–	10	9	Square (Sq)	PDMS	RM with photoresist (PR) mold	National Institutes of Health 3‐day transfer, inoculum 3 (NIH 3T3)	CTF measurements during mechanical stimulations through nanowire‐embedded magnetic micropillars	Fibronectin (FN)/Pluronic F127	Fluorescence imaging DiI	[136]
3	11	9	Sq	PDMS	RM with PR mold	MEF, HUVEC, MCF10a, SMC	To study the shear force geometry at the cell–matrix interface	FN/Pluronic F127	Fluorescence imaging	[128]
3	11	9	Sq	PDMS	RM with PR mold	NIH 3T3	Introduction of micropillar arrays as a CTF measuring technique	FN/collagen IV	Fluorescence imaging	[15]
2–3	6–10	6–9	Sq	PDMS	RM with PR mold	Stem‐cell‐derived cardiomyocytes	Contractile force measurement of Stem cell‐derived cardiomyocytes (SC‐CMs)	Pluronic F‐127	Fluorescence imaging Alexa Fluor 594	[160]
2	51	–	Within 6 μm groves	Si pillars and SiO_2_ attachment pads	DRIE	Endothelial cells/fibroblasts	Geometry‐induced contact guidance and CTF	–	–	[14]
2	5	3	Sq	PDMS	RM	NIH 3T3	Study of CTF correlated with nuclear dynamics	FN/Pluronic F‐127	Bright‐field imaging	[168]
3,4	9	9	Sq	PDMS	RM	SMC	CTF measurements while mechanical stimulations through magnetic nanoparticle embedded micro posts	FN/Bovine serum albumin (BSA)	Bright‐field imaging	[147]
10	–	20–30	Sq	PDMS	RM with PR mold	Cardiomyocytes	Study of CM contraction while mimicking 3*d* cell morphology by suspending cells between two pillars	–	Fluorescence imaging DiD	[149]
40/80 nm	4	1	Hexagonal (Hex)	GaP	MOVPE	Nerve cells	pN scale force measurement of neural growth cones	Laminin	Fluorescence imaging Alexa Fluor 594	[99]
≈2 and ≈1 elliptical	4.7, 3.7	4	Hex	PDMS	RM	MDCK epithelial cells	CTF and anisotropy mediated cell growth and migration	FN/Pluronic F127	Fluorescence imaging Cy3	[170]
2	1–12	4	Hex	PDMS	RM with DRIE mold	hMSCs	Substrate rigidity mediated stem cell differentiation. Pillar height was varied for rigidity modulation.	FN, collagen type I	Fluorescence imaging Δ9‐DiI	[19]
1,2	2,7	2	Hex	PDMS	RM with DRIE mold	MDCK	Study of traction forces induced by epithelial cell monolayers	FN	Bright‐field and fluorescence imaging	[33]
≈2(1.83)	≈1−15(0.97–14.7)	4	–	PDMS	RM with DRIE mold	Stem cells	Study of substrate stiffness on stem cell differentiation, cell morphology, and CTFs	FN/Pluronic F127	Fluorescence imaging Dil	[17]
1.83	9	4	Hex	PDMS	RM with DRIE mold	HUVEC	Flow‐induced CTF in vascular endothelial cells (pillar array located inside a flow chamber)	FN/Pluronic F127	Fluorescence imaging	[164]
300–800	4–5 mm	4 mm	2 pillars in a well	PDMS	RM with Teflon mold	Mouse myoblasts tissues	Screen the effect of certain drugs on tissue contractile force	–	–	[150]
3	10	9	Sq	PDMS, Co nanowires suspended	RM	NIH 3T3	FA and CTF response to external forces induced by magnetic wire embedded micro posts	FN/Pluronic F127	Fluorescence imaging Δ9‐DiI	[203]
3	10	9	Sq	PDMS	RM with PR mold	NIH 3T3	Study relationship between CTF and FN fibril growth at cell–matrix interface	FN/Pluronic F‐68	Fluorescence imaging Alexa Fluor 647	[169]
3	9	6	Sq	PDMS	RM	NIH 3T3 and human osteosarcoma cells	To study the cell migration associated with CTF in confined 3*d* spaces. The pillar array is placed inside confined microchannels.	Collagen type I/Pluronic F127	–	[139]
1–2	1.6–6	–	Hex	PDMS	RM	MDCK	To study the CTF mediation based on substrate rigidity	FN	Fluorescence imaging	[124]
1–2	3,4	3–8	Hex	PDMS	RM with DRIE	MDCK	CTF mapping in collective cell migration of MDCK monolayers	FN	Bright‐field and fluorescent Alexa Fluor 488	[18]
3	9	9	Hex	PDMS	RM	SMC	Study of changes in cell‐generated traction forces while microscopic cell stretching	FN	Bright‐field imaging	[125]
1	4 and 9	5	Sq	PDMS, poly lactic‐co‐glycolic acid, Flexistene	RM	NIH 3T3	Cell migration in adjacent micropillar arrays with different stiffnesses	FN	–	[34]
1,2 elliptic pillars with minor axis ‐ 1 and major axis ‐ 2	1.4,7 respectively 3,6	–	Hex	PDMS	RM with DRIE	3T3 fibroblastic cells and MDCK	Study CTF migration response on substrate mechanical properties. One array consists of elliptical micropillars to study the effect of substrate anisotropic rigidity.	FN/methoxy polyethylene glycol maleimide	Bright‐field/florescence Cy3	[148]
2	3.3–4.7	4	Hex	PDMS	RM with DRIE	Vascular endothelium cells	Cell traction force changes induced in neutrophil transmigration through endothelium monolayers	FN/Pluronic F127	Fluorescence microscopy Alexa Fluor 488	[166]
5 3	25	7 5	Sq	PDMS	RM with DRIE	Vascular smooth muscle cells	Study CTFs on micropillar substrates with different rigidities	Collagen or FN	Phase contrast microscopy	[133]
1.8	–	4	Hex	PDMS	–	NIH 3T3	Characterization of mechanical aspects of actomyosin cortex and stress fiber network – Magnetic wire is embedded for external magnetic stimulations.	FN	Bright‐field imaging	[142]
–	–	–	Hex	PDMS	RM with DRIE	Dendritic cells	Traction forces associated with dendritic cell motility induced by chemotaxis	FN/Pluronic F127	Fluorescence imaging Dil	[171]
≈0.8 ≈1.83	≈2.48 ≈5.7	2 4	Hex	PDMS	RM	MEFs, hMSCs	Assessing the impact of cell adhesion area on CTFs and cell spread. Pillar arrays with two different pillar diameters were utilized.	FN/Pluronic F127	Alexa Fluor 647	[29]
3	13.4	6 bottom array 6–10 top array	Hex	PDMS	RM	MC3T3‐E1	CTF while cells migrate in a 3D confined microenvironment. Cells were seeded in between two micropillar arrays facing each other.	FN/Pluronic F127	Laser scanning confocal microscopy. Dil	[179]
2.7 (280 nm on micropillars)	12 (nanopillar height – 500 nm)	3.3 edge to edge (nano pillar 280 nm)	Hex	PDMS	RM with multistep photolithography and DRIE	MC3T3‐E1	Study the effect of surface coating of micropillar top surface on CTFs. Four pillar arrays with top surfaces covered with nanopillars, TiO_2_, SiO_2_, and plain PDMS were used.	FN/Pluronic F127	Fluorescence imaging Dil	[137]
3	13.4	6–8	Hex	PDMS	RM with SU8	MC3T3‐E1	Study cell migration and associated CTFs in confined channels. Pillars are placed inside enclosed channels.	FN/Pluronic F127	Dil—confocal microscopy	[126]
≈22	≈50	450,770, 1600 pillars per 1 mm^2^	Sq	Hydrogel	UV stereolithography (custom setup)	Mesenchymal stromal cells	Study 3D effects extracellular environment mechanical properties by seeding cells inside pillar arrays with different pillar densities	–	–	[238]
Sq pillar topology	≈6	–	Sq	PDMS	RM	Human pulmonary artery endothelial cells	Traction forces changes associated with transendothelial migration through endothelial monolayers	FN/Pluronic F127	Fuorescence imaging Dil	[167]
3	9	9	Sq	PDMS	RM with PR mold	Porcine aortic smooth muscle cells	Changes in traction forces and focal adhesions during SMC contraction	FN/BSA	Bright‐field imaging	[138]
7.6 × 7.7 3.9 × 7.8 5.7 × 5.7 3.7 × 5.7 sq pillars	–	–	Sq	PDMS	RM with soft lithography	hMSCs and cardiomyocytes	Study of cell elongation and morphology on anisotropic 3D confinement. Cells are seeded inside the anisotropic micropillar array.	Pluronic F127 on pillar tops FN on sides	–	[239]
3.5 ± 0.5	13 ± 1	8 ± 1	Sq	Polyethylene glycol diacrylate	Custom vat photopolymerization lithography setup	Rat embryonic fibroblasts (REF)‐expressing yellow fluorescent protein (YFP)‐fused to paxillin (PAX) cells	Contribution of *α*_v_ *β*_3_ and *α*_5_ *β*_1_ Integrin on cell‐generated traction forces at FAs	Nanogold particles	Bright‐field and fluorescence imaging	[142]
5 × 5 20 × 20 Sq pillar	10	10 45 70 220	Sq	Vertically aligned multi‐wall carbon nanotubes	CVD	Chondrocyte	Inducing unidirectional growth of chondrocytes by mimicking the 3D environment and rigidity of cartilage's ECM	–	–	[108]
27	105	80	Hex	PDMS	RM with PR mold	Neonatal rat cardiomyocyte, human embryonic stem‐cell‐derived cardiomyocytes	Contraction force analysis while cells are suspended between pillars. A thermo‐responsive sacrificial layer restricted cell adhesion to pillar tops.	Laminin/poly (N‐isopropyl acrylamide) sacrificial layer	–	[135]
150 nm	1.5	800 nm	Sq	InGaN/GaN	Dry etching of GaN thin film using NI metal mask	Cardiomyocytes	Real‐time traction force analysis in CM beating	–	Fluorescence imaging—photoluminescence (PL) from the piezo‐phototronic effect	[146]
2	6	4	Sq	PDMS	RM with PR mold	Mouse embryo fibroblasts	Assess the viability of using moire fringe patterns created by a double‐sided micropillar array in CTF measurements.	FN	Distortions of moire fringe pattern with a double‐sided array	[144]
2	6	4	Sq	PDMS	RM with DRIE	Vascular smooth muscle cells	Assess the viability of using moire fringe patterns in pillar deflection measurements using two pillar arrays.	Laminin	Distortions of moire fringe pattern—two misaligned arrays	[143]
2	3.4	4	Hex	PDMS	RM with DRIE	C2 mouse myogenic cells	Measurement of traction force transferred through N‐cadherin contacts	FN/BSA	Bright‐field imaging	[351]
1,2	–	–	Hex	PDMS	–	REF52 fibroblast	To study the relationship between CTF and FA size with substrate stiffness	FN	Fluorescence imaging	[30]
90 ± 5 nm GaP segment 105 ± 5 nm GaIP segment	≈2.6 μm ≈0.4 μm	2 μm^−2^ density	Hex	GaP	Metal‐organic vapor phase epitaxy	MCF7 breast cancer cells, MCF10A normal‐like breast epithelial cells	CTF as a biomarker for cancer cell identification and the effect of certain anticancer drugs on CTFs	–	Near infrared photoluminescence of gallium indium phosphide (GaInP) tips	[140]
2	4	4	Sq	PDMS	RM with DRIE	Cardiomyocytes	Contraction force measurement and optimizing interpillar distance to avoid cell contact with the substrate bottom surface	Lamine	Phase contrast microscopy	[31]
2	7	5	Sq	PDMS	RM with PR mold	Human airway smooth muscle, rat aortic smooth muscle cells	Cell‐generated traction force measurement and optimizing the design of the micropillar substrate	Collagen I	Fluorescence imaging CdSe/CdS quantum dot labels	[134]
20	90	–	–	PDMS	RM with PR mold	Neonatal cardiomyocytes	To study the stability of CM binding to the pillars through laminin and contractile phenotypes	Laminin	Fluorescence and bright field Alexa Fluor 488	[352]
2.3	7.2	6	Sq	PDMS	RM with PR mold	Pluripotent stem‐cell‐derived cardiomyocytes	Contractile performance of hiPSC–CMs	Laminin, FN, collagen IV/Pluronic F‐127	Alexa Fluor 594	[158]
1	5	3 × 3 3 × 6 3 × 9 3 × 12	Rectangular anisotropic array	Si	DRIE	Hippocampal neurons	Directional neurite growth on anisotropic pillar arrangement	–	–	[230]
1.6	3	4,1,8,1,4,0.6 (edge to edge) 1.6 *x* direction, 0.6–5.6 *y* direction	Hex, anisotropic rectangular	PDMS	RM	Hippocampal neurons	Chemical and topographical‐signal‐induced growth of neurite	–	–	[227]
10 Sq & Cr pillar topologies	10	10	Sq	PDMS	RM with PR mold	Cortical interneurons	Influence of substrate topography on cell morphology and migration	N‐cadherin and laminin/BSA	–	[231]
200 nm 2	400 nm 2	–	Sq	PDMS	Imprint lithography with Si mold	hMSC MCF7 COS7	Influence of pillar/substrate topography on cellular endocytosis process	–	–	[49]
500 nm 2	2 10	2 4	Sq	Polycarbonate	Nanoimprinting	C17.2 neural stem cells	To study the effect of different micropillar topologies on cellular response	–	–	[195]
Top 2 Bottom 2–7	7	10	Sq and Hex	PDMS	RM with double exposed PR mold	NIH 3T3	Cell migration on stepped micropillars with varying based diameter. Base diameter gradient creates a stiffness gradient in micropillars.	FN/Pluronic F127	Fluorescence imaging	[173]
600 nm	900 nm	3.6 and 4	Hex with two lattice distances	Si	DRIE	MC3T3‐E1	Cell morphology and spreading on hexagonally arranged micropillars with two different inter‐pillar spacings	–	Fluorescence imaging and SEM	[228]
Top 800 nm Bottom 15 nm	10	2–6	Hex Multiple arrays with different spacing	GaAs	–	Dictyostelium discoideum	Researchers examined cellular adhesion forces up to 50 pN using these conically shaped nanopillars that taper from top to bottom.	–	Fluorescence confocal imaging	[196]

a) In this table, all dimensions are presented in micrometers and micropillar topology is circular unless otherwise specified. The acronyms Sq and Hex denote square and hexagonal shapes, respectively. In fabrication procedures, RM signifies replica molding, and PR indicates photoresist. Regarding cell types, MEF, HUVEC, SMC, and hMSC refer to mouse embryonic fibroblast, human umbilical vein endothelial cells, smooth muscle cells, and human mesenchymal stem cells, respectively. All other cell names correspond to their standard nomenclature, such as NIH 3T3. Other acronyms used in the table are defined at their first use.

### 2.1 Fabrication

#### 2.1.1 Replica Molding/Soft Lithography

**Figure 3:**
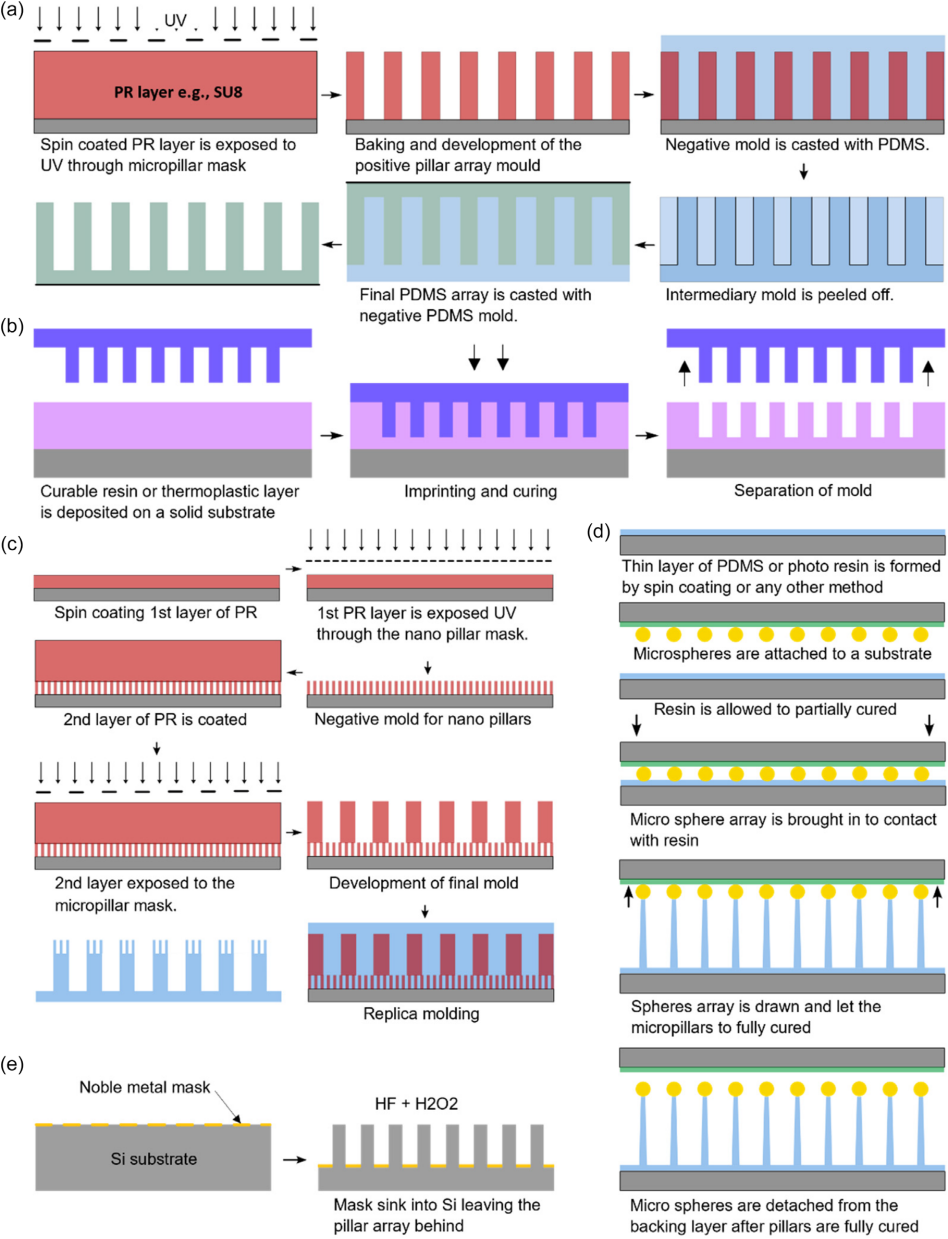
Micropillar array fabrication processes. a) General process of PDMS replica molding using a photoresist. If the negative mold is directly fabricated from DRIE or a similar technique, only the last two steps of this process are required. b) Process of imprint lithography. If a thermoplastic is used, heat is applied at the imprinting step. In UV resins, transparent imprint mold is used to allow exposure to UV through the mold. c) Fabrication of nanopillars on top of micropillars using two‐step photolithography. By combining negative and positive photoresists in multistep lithography, hierarchical features such as pillars with different heights can be fabricated. Reproduced with permission.^[^ ^35^ ^]^ Copyright 2009, John Wiley and Sons. d) Direct drawing of micropillars. Reproduced under terms of CC BY‐NC 4.0.^[^ ^52^ ^]^ Copyright 2017, The Authors. Published by Scientific Reports. e) Metal‐assisted chemical etching (MacEtch). A noble metal mask sinks into the Si, acting like a negative photoresist.

Replica molding is the most popular fabrication technique for mechanobiology‐related micropillar production. Replica molding offers a relatively inexpensive and flexible method for polymer microstructure fabrication. In this method, a master mold is used to transfer a design into a castable or formable material. If the mold is negative with an array of holes, the final array can be directly cast, and if it is a positive mold, casting is done through an intermediate PDMS mold (refer to **Figure** **3**a).^[^ ^15^ ^]^

Photolithography is a commonly employed technique for fabricating micropillar array molds on a photoresist (e.g., SU‐8) or silicon substrate for soft lithography. While versatility and capability of fabricating nanoscale structures are advantages of photolithography, it can suffer from light diffraction, reducing precision and verticality of walls in high aspect ratio micropillars. The negative mold can be directly fabricated on silicon by using deep reactive ion etching (DRIE) to mitigate these effects.^[^ ^33^ ^]^ Multistep photolithography can be employed to fabricate micropillar molds with more complex hierarchical features, such as varying height pillar arrays.^[^ ^34^ ^]^ Greiner et al. demonstrated the mold fabrication of 5 μm pillar arrays on top of micropillars with a diameter of 50 μms using two‐step photolithography (see Figure 3c).^[^ ^35^ ^]^ Additive manufacturing is also evident in fabricating molds for more complex pillar geometries, such as bent pillar ends.^[^ ^36^ ^]^ As a more straightforward manufacturing method, laser‐machined poly(methyl methacrylate) can be used for molding.^[^ ^37^ ^]^ However, the use of this method is only evident in relatively larger micropillars.^[^ ^38^ ^]^

PDMS stands out as the most popular material used with replica molding and cell mechanobiology due to its excellent biocompatibility, ability to replicate nanolevel features, and simpler fabrication procedures.^[^ ^39^ ^]^ With most mammalian tissues exhibiting an elastic modulus below 1 MPa,^[^ ^40^ ^]^ tunable elastic modulus of PDMS, ranging from 100 kPa to 3 MPa, makes it more compatible with biological tissues compared to metals like silicon with GPa range elastic modulus, which is frequently used in micromanufacturing. This adaptability allows for comparatively thicker pillars with PDMS, providing increased cell adhesion area Despite its widespread use, PDMS has several limitations that constrain its applicability in some instances. Its inherent hydrophobicity restricts direct cellular attachment to micropillars, necessitating surface treatments or chemical coatings before cells can be cultured.^[^ ^41^, ^42^ ^]^ Additionally, PDMS has a tendency to absorb small molecules, which can interfere with the experiments such as drug delivery, through unwanted surface binding.^[^ ^43^ ^]^ Although PDMS provides a tunable elastic modulus suitable for most cell types, it may not be appropriate for very soft tissues, like mucosa and brain tissue, which have sub‐kilopascal stiffness, or for stiffer tissues, like cartilage and bone, which exhibit higher stiffness values that PDMS cannot match.^[^ ^44^, ^45^, ^46^ ^]^ Hydrogel is an alternative material which used in micropillar fabrication to achieve softer micropillars.^[^ ^42^, ^47^ ^]^ Similar replica molding techniques are used for hydrogel micropillar fabrication.^[^ ^32^ ^]^ For applications where higher micropillar stiffness is required, poly(lactide‐co‐glycolide) (PLGA) is another commonly used polymer. PLGA, stiffer than PDMS, is often employed in mechanobiology studies where pillars do not need to be flexible.^[^ ^48^ ^]^

Imprint lithography emerges as a variant of replica molding suitable for micro/nanopillar fabrication.^[^ ^49^ ^]^ Imprint lithography can be utilized with PDMS, UV–curable polymers, and thermoplastics. Following a similar approach to soft lithography, an initial negative mold must be fabricated using any of the aforementioned methods. The negative mold is then pressed onto either an uncured polymer or a heated thermoplastic layer coated on a rigid substrate, followed by curing and demolding (refer to Figure 3b). In addition to micro/nanopillars, Jeong et al. utilized this method to fabricate nanopillars atop replica‐molded PDMS micropillars.^[^ ^50^ ^]^ They molded the micropillar array before it was fully cured, then pressed a negative silicon oxide mold with nanoholes. Recent advancements and techniques in imprint lithography have been reviewed in detail elsewhere.^[^ ^51^ ^]^

The direct drawing of micropillars from PDMS introduces a novel manufacturing technique uniquely tailored for micropillar fabrication. This method excels in producing micropillars with aspect ratios exceeding 100. Initially, a solid substrate is coated with PDMS, which is then allowed to cure partially. Subsequently, a microsphere is brought into contact with the partially cured PDMS, allowing it to adhere. The sphere is then drawn vertically upward, creating a PDMS micropillar. To solidify the micropillar, the polymer is cured further using either UV light or heat, see Figure 3d.^[^ ^52^ ^]^ An array of micropillars can be fabricated by employing a patterned microsphere array.^[^ ^53^ ^]^ This method offers excellent control over the height of the micropillars. However, the pillars’ diameter may vary slightly from bottom to top.

#### 2.1.2 Lithography

Direct fabrication of micropillars on a silicon substrate with photolithography followed by certain etching processes, such as reactive ion etching, is possible. However, in most cellular force sensing applications, these silicon pillar arrays are used as molds for replica molding.^[^ ^28^ ^]^ Compared to polymers like PDMS, silicon has a very high Young's modulus. To achieve pillar rigidities suitable for CTF sensing, silicon pillars must have lesser diameters and very high aspect ratios compared to PDMS counterparts. DRIE is an extension of reactive ion etching, capable of producing high aspect ratio structures with vertical walls.^[^ ^54^ ^]^ The use of DRIE to fabricate silicon micropillars with aspect ratios of more than 1:20 is well documented in the literature.^[^ ^14^, ^55^ ^]^ These micropillars have considerably less top surface area than PDMS pillars. Thus, wider attachment pads can be fabricated on pillar tops to increase focal adhesion area.^[^ ^56^ ^]^

Metal‐assisted chemical etching (MacEtch) is a relatively new process that emerged in the 2000s, capable of producing high aspect ratio nanopillars through wet etching.^[^ ^57^ ^]^ In MacEtch, part of the silicon substrate that needs to be etched away is covered with a noble metal such as Ag, Au, Pt, or Pd, and the substrate is subjected to an etchant consisting of hydrogen fluoride (HF) and an oxidant. Silicon beneath the noble metal mask etches faster, sinking the noble metal layer into the silicon, creating a cyclic process (refer to Figure 3e). In this process, noble metal acts like a negative resist mask by catalyzing the etching step.^[^ ^58^ ^]^ Researchers have used MacEtch to fabricate ciliated micropillars. They fabricated micropillars with DRIE and deposited silver nanoparticles on the micropillar side surfaces with reverse pulse plating. Locations of silver particles were etched with MacEtch up to 400 nm depth on side walls using HF and H_2_O_2_, leaving a hairlike nanostructure on the micropillar surface.^[^ ^59^ ^]^

#### 2.1.3 Inkjet Printing

**Figure 4:**
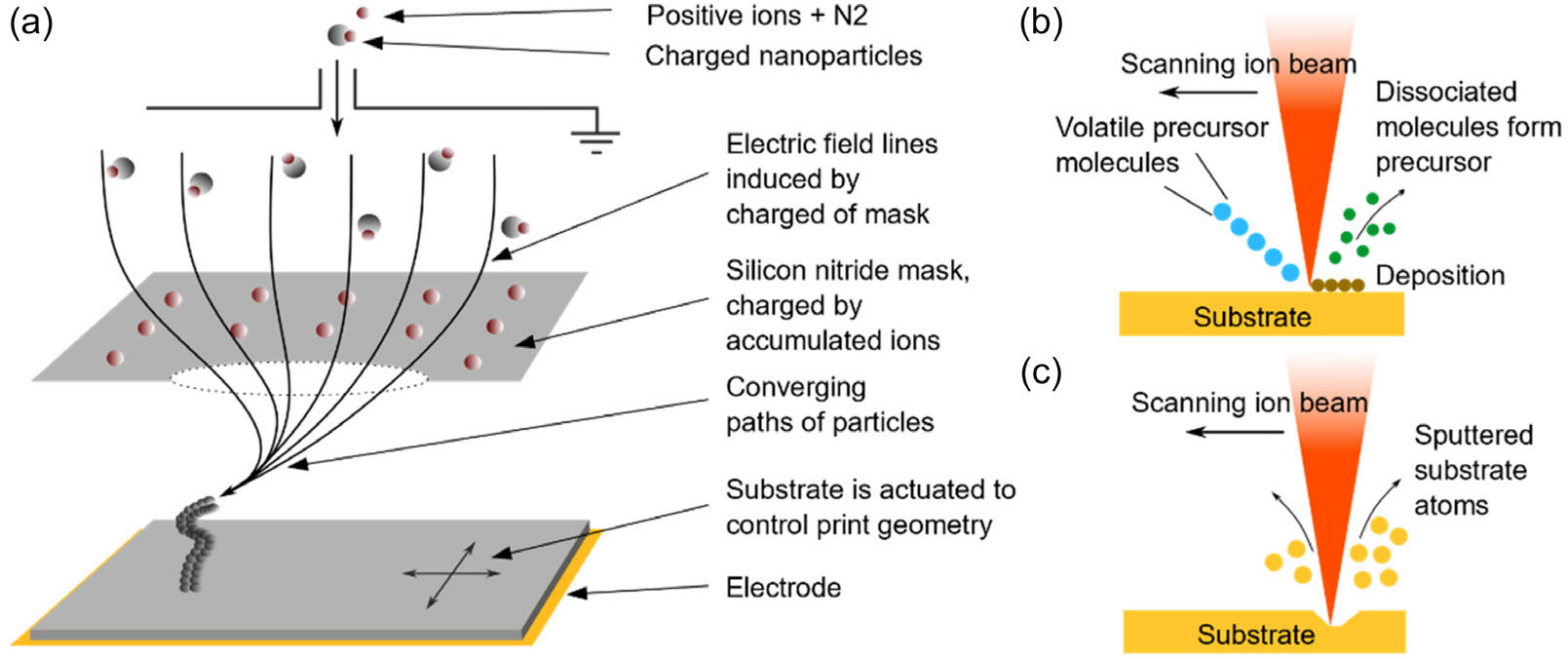
Rapid fabrication techniques from CAD models. a) Static electric‐field‐assisted aerosol jet rapid prototyping. An electric field guides the charged nanoparticles. This method can print overhanging microstructures without external support structures. Reproduced with permission.^[^ ^65^ ^]^ Copyright 2021, Springer Nature. b) Focused‐ion‐beam‐induced deposition. A volatile precursor is dissociated and deposited on the substrate under the excitation of an ion beam. The mechanism of electron‐beam‐induced deposition is also similar. c) Focused ion beam milling. When the energy of ions exceeds the substrate surface binding energy, substrate atoms are sputtered from the workpiece.

Inkjet printing emerges as a versatile technique compatible with a wide range of materials, including polymers, metals, and ceramics.^[^ ^60^ ^]^ The utilization of inkjet printing^[^ ^61^ ^]^ and multi‐jet printing^[^ ^36^ ^]^ for micropillar fabrication is well documented in the literature. In its basic form, liquid ink or particles suspended in a solution are utilized, with a thermal or piezoelectric actuator controlling droplet generation, followed by natural or forced curing.^[^ ^62^ ^]^ Aerosol jet printing represents a variant of inkjet printing capable of achieving higher resolutions.^[^ ^60^ ^]^ Azahar et al. employed this technique for nanoparticle 3D printing to fabricate a hollow micropillar array with silver electrodes for biomolecule sensing.^[^ ^63^ ^]^ In aerosol jet printing, liquid ink or ink with suspended nanoparticles is ultrasonically atomized, mixed with an inert gas for transportation, and dispensed onto a heated substrate in the intended pattern.^[^ ^64^ ^]^ Jung et al. experimented with a further developed version of this method.^[^ ^65^ ^]^ They generate positively charged aerosol particles and ions with an electrical discharge. The print substrate is negatively charged, and a dielectric mask with holes is placed between the print substrate and the aerosol jet (refer to **Figure** **4**a). Ion accumulation around the holes functions as electrostatic lenses. It focuses aerosol jets like a nozzle, allowing this technique to print high‐resolution complex overhanging structures.

#### 2.1.4 Vat Photopolymerization

Vat photopolymerization comprises a set of techniques that utilize controlled photopolymerization of a resin to fabricate both macro‐ and micro‐3D structures layer‐wise. Based on the curing method, these techniques are classified into stereolithography, digital light processing, continuous digital light processing, and a few other variants.^[^ ^66^ ^]^ Microstereolithography represents an advancement of conventional stereolithography for microstructure fabrication. In conventional stereolithography, a polymerizing laser point scans the pattern on a photo‐curable resin using variable focusing optics and galvanometric mirrors. Conversely, the laser point remains stationary in microstereolithography, and the printing stage translates in the *x* and *y* directions.^[^ ^67^ ^]^ This modification eliminates the resolution limitations associated with dynamic optics, although it results in a slower process than the conventional method. Using this technique, studies have demonstrated the fabrication of micropillars as small as 100 μm with aspect ratios exceeding 10.^[^ ^68^ ^]^ Projection microstereolithography is a further advanced version of stereolithography, which combines digital light processing techniques to project an entire layer onto the resin surface in a single exposure.^[^ ^69^ ^]^ This approach significantly speeds up the process and allows for the fabrication of micropillars as small as 30 μm with aspect ratios greater than 30, as reported in the literature.^[^ ^70^ ^]^ Evidently, this technique can reach up to a resolution of 500 nm.^[^ ^71^ ^]^ Photopolymerization techniques are also evident in hydrogel micropillar fabrication.^[^ ^72^ ^]^ In addition to hydrogels alone, studies have utilized cell‐laden bio‐ink polymerization with visible light to fabricate micropillars that mimic intestinal villi.^[^ ^73^ ^]^ By controlling the light source exposure time, researchers could tune Young's modulus of pillars over a broad range.^[^ ^42^ ^]^ Recent advancements in vat photopolymerization techniques and current challenges and future perspectives are reviewed elsewhere.^[^ ^74^ ^]^

Two‐photon polymerization (2PP) is a highly flexible photopolymerization‐based 3D‐printing process that enables the fabrication of polymer micropillars with resolution exceeding 100 nm.^[^ ^75^, ^76^, ^77^ ^]^ In the 2PP process, photoinitiators initiate resin polymerization by absorbing two photons with half of the energy required for excitation. The 2PP uses femtosecond infrared lasers, which do not have enough photon energy for single‐photon polymerization. Thus, the 2PP process will take place at the tightly focused point of the laser beam, where one photoinitiator has the highest possibility of receiving two photons. This enables its capability to create actual 3D structures within a resin.^[^ ^77^ ^]^ The use of 2PP is evident in fabricating complex micropillar geometries, such as stepped micropillars with varying base dimensions for cell migration studies.^[^ ^78^ ^]^

#### 2.1.5 Electrochemical Deposition

Electrochemical deposition has been a longstanding method for plating metals onto conductive substrates. The deposition area must be precisely regulated to fabricate 3D structures with this technique.^[^ ^79^ ^]^ Klösel et al. discussed using a patterned photoresist layer as a template for micropillar fabrication with electrochemical deposition.^[^ ^80^ ^]^ An anodic aluminum oxide is a form of aluminum oxide known for forming porous structures with parallel nanopores. The parameters of the anodizing process enable control over pore diameter, interpore distance, and pore geometry.^[^ ^81^ ^]^ Chen et al. demonstrated the fabrication of a CoFe_2_O_4_ nanopillar array using an anodic aluminum oxide template with electrochemical deposition.^[^ ^82^ ^]^ A copper layer, to serve as the cathode, was deposited on the anodic alumina template using electro‐beam evaporation. Yanagishita et al. etched an anodic alumina layer using a patterned mask to fabricate a template for relatively larger, high aspect ratio Ni micropillars with vertical walls.^[^ ^83^ ^]^ For the electrochemical deposition of Ni, Zn seeds were deposited inside the microcavities as electrodes. Localized electrochemical deposition denotes fabrication processes based on electrochemical deposition without templates.^[^ ^84^ ^]^ Xu et al. provided a detailed review of maskless localized electrochemical deposition techniques.^[^ ^85^ ^]^ Due to their fabrication flexibility, they are often considered a 3D‐printing technique.

LIGA (LIGA—Lithographie, Galvanoformung, and Abformung) is another fabrication technique based on electrochemical deposition, originally utilizing synchrotron radiation lithography to produce templates.^[^ ^86^ ^]^ UV LIGA, a more accessible fabrication process,^[^ ^87^ ^]^ is also used for micropillar fabrication,^[^ ^88^ ^]^ although it is not widely documented in the literature.

#### 2.1.6 Chemical Vapor Deposition

Chemical vapor deposition (CVD) is conventionally used to deposit high‐quality thin films on a substrate via a chemical reaction of a gas precursor.^[^ ^89^ ^]^ Variants of CVD that allow control over deposition sites have been utilized to fabricate extremely high aspect ratio micro/nanopillar arrays.^[^ ^90^, ^91^ ^]^ By controlling the growth sites’ crystallographic properties, the pillars’ cross‐sectional shape can also be modulated.^[^ ^92^ ^]^ Metal‐organic vapor‐phase epitaxy, a CVD technique commonly employed in semiconductor fabrication, has been adapted for nanopillar fabrication for a wide range of biosensing applications.^[^ ^93^, ^94^, ^95^, ^96^ ^]^ It employs vaporized organic–metallic precursors with a neutral carrier gas, and upon contact with the heated substrate, they decompose and deposit onto the substrate.^[^ ^97^ ^]^ In micropillar fabrication using metal‐organic vapor‐phase epitaxy, preferential growth sites must be predefined on the substrate with seed particles.^[^ ^98^ ^]^ This technique has been used to grow high‐density gallium phosphide (GaP)^[^ ^99^ ^]^ and indium phosphide (InP)^[^ ^100^ ^]^ nanopillar arrays, which provide high‐resolution CTF measurements. Additionally, CVD‐fabricated pillars have been used to mimic and modulate ECM properties, with parameters like seed particle size and growth time being adjusted to control pillar characteristics.^[^ ^101^ ^]^ These experiments defined growth sites with gold particles deposited through electron beam lithography or nanoimprinting.

Focused electron‐beam‐induced deposition and focus‐ion‐beam‐induced deposition stand as two other CVD techniques capable of fabricating high‐resolution nanopillar structures.^[^ ^102^, ^103^ ^]^ Both techniques rely on the adsorption of volatile precursor molecules onto a substrate and their dissociation into the final material under the excitation of a focused electron or ion beam, see Figure 4b.^[^ ^104^ ^]^ Commonly, metal‐organic precursors are used with these processes, resulting in the final structure comprising an amorphous carbon matrix embedded with metal nanocrystals.^[^ ^105^ ^]^

CVD is also employed in the fabrication of carbon‐based structures, diamond micropillars, and vertically aligned carbon nanotube arrays. In diamond micropillar fabrication, diamond nanocrystals are seeded on a silicon wafer, and the pillar array is grown with CVD using a guiding mask such as anodic aluminum oxide or patterned Si.^[^ ^106^ ^]^ Due to superior mechanical properties, diamond pillar arrays are usually used as master molds for polymer micropillar fabrication. Carbon nanotube arrays can also be synthesized by arc discharge and laser ablation, though CVD is most utilized in experiments.^[^ ^107^ ^]^ In the CVD technique, pillar locations are marked with a catalyst and a hydrocarbon is used as the precursor. At sufficiently high temperatures, the hydrocarbon is decomposed, and vertically aligned carbon nanotubes are grown at catalyst‐marked locations.^[^ ^108^ ^]^ The biological application of vertically aligned carbon nanotube arrays is comprehensively reviewed elsewhere.^[^ ^109^ ^]^

#### 2.1.7 Focused Energy Beam Machining

Removing material directly with focused energy beams is widely used in both macro‐ and microfabrication. Techniques such as laser beam machining, electron beam lithography, and focused ion beam milling are among the most popular in this category. Due to maskless fabrication procedures, these techniques allow rapid production of structures directly from computer‐aided design drawings. The use of laser beam machining in fabricating micro/nanopillar‐like structures on various materials is well documented in the literature.^[^ ^110^, ^111^ ^]^ Generally, these techniques are used to remove material from the substrate. However, micropillars taller than the original substrate top surface can be fabricated with precise control over the recast layer.^[^ ^112^ ^]^ Apart from the direct fabrication of micropillars, laser machining can also be used for mold fabrication.^[^ ^37^ ^]^

Electron beam lithography is a process extensively used in electronic micromanufacturing, offering significant potential for high resolution owing to the sub‐nanometer wavelength of electron beams.^[^ ^113^ ^]^ Hallstrom et al. used this technique to define nanopillar locations with gold for epitaxial growing.^[^ ^99^ ^]^ Focused ion beam milling is another maskless top‐down process capable of manufacturing high‐resolution nanostructures directly on silicon. Traditionally, a gallium liquid metal ion source is used as the ion source, and the focused beam scans the removal pattern. Surface atoms of the substrate will be sputtered once Ga^+^ ions exceed the surface binding energy, see Figure 4c.^[^ ^114^ ^]^

Despite the advantages of energy‐beam‐based direct machining processes, they can introduce several defects and mechanical property changes, such as heat‐affected zones to the materials.^[^ ^115^ ^]^ Comprehensive reviews on recent advances and techniques of laser beam machining,^[^ ^116^, ^117^ ^]^ electron beam lithography,^[^ ^118^ ^]^ and focused ion beam milling^[^ ^119^ ^]^ are available in the literature.

## 3 Micropillar Deflection‐Based Sensing

Micropillar‐deflection‐based force sensing stands out as the most unique application of micropillars. This section delves into the pillar array functionalization, characterization for cellular force sensing, and the various applications discussed in the literature. The micropillar functionalization procedure is common for micropillar‐based ECM modulation as well.

### 3.1 Micropillar Functionalization

Post‐processing may be required based on the application after the fabrication of micropillars. In experiments measuring CTF, pillar top surfaces need to be functionalized for cell adhesion. Focal adhesions are multi‐protein structures that mediate the force transfer between cells and ECM.^[^ ^120^ ^]^ Focal adhesions consist of integrins, the primary receptor proteins, which bind the cell's cytoskeleton to most ECM proteins, including fibronectin (FN), laminins, and collagens.^[^ ^121^ ^]^ In vitro, ECM proteins are artificially absorbed onto the surface of the growth substrate to mediate focal adhesions between the cell and the growth substrate. Different ECM proteins have different effects on cellular response. For example, studies on pluripotent stem cells suggest that fetal bovine serum most effectively supports cell propagation while FN and laminin promote differentiation.^[^ ^122^ ^]^

**Figure 5:**
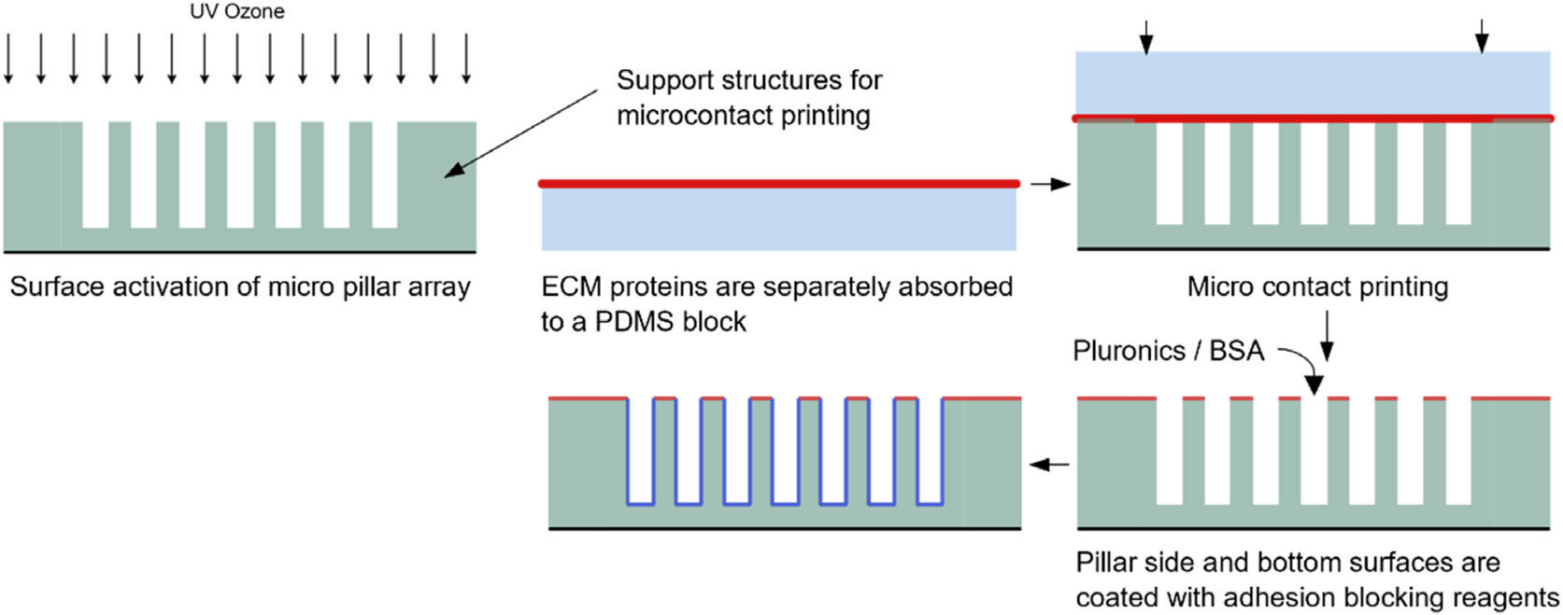
Process of micropillar functionalization for cell seeding. In microcontact printing, support structures around the micropillars are critical to avoid pillar collapse under contact.

PDMS micropillars are typically hydrophobic, and their surface needs to be modified/activated before the absorption of ECM proteins.^[^ ^123^ ^]^ Surface activation usually involves UV–ozone oxidization or oxygen plasma treatment. Microcontact printing is the most popular way of ECM protein deposition, see **Figure** **5**. A separate PDMS stamp is prepared in microcontact printing, and protein is first absorbed into the stamp. Then, the stamp is brought into contact with the pillar array to allow protein transfer to pillar tops.^[^ ^24^ ^]^ However, in some studies, the whole array was directly immersed in ECM proteins as surface tension prevents protein absorption on pillar side surfaces.^[^ ^124^ ^]^ If pillars need to be labeled, either fluorescently tagged ECM proteins are used or stained with the dye following the microcontact printing. Finally, the pillar side surfaces, and bottom surfaces are coated with nonadhesive reagents such as Pluronic f‐127 or bovine serum albumin (BSA) to restrict cell adhesion to pillar tops.^[^ ^125^, ^126^ ^]^

### 3.2 Pillar Characterization for Force Sensing

**Figure 6:**
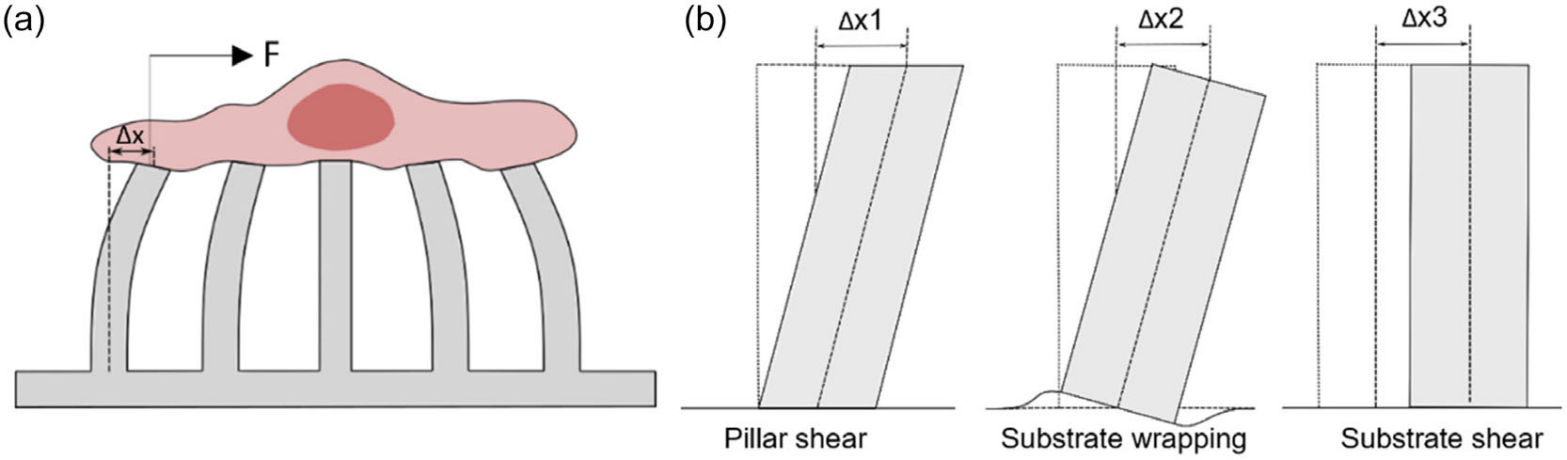
Micropillar deflection geometries. a) Geometry of the ideal CTF on micropillars. b) Additional components of pillar top displacement, which are not considered in beam‐theory‐based characterization. When Δx is measured, Δx1, Δx2, and Δx3 are included in the measured value in addition to the pure bending component. Δx3 can be removed by comparing pillar top displacement with pillar bottom. However, separating Δx1 and Δx2 from Δx is difficult. Reproduced with permission.^[^ ^129^ ^]^ Copyright 2010, American Chemical Society.

Considering the cell attachment to the pillar top surface, the traction force along the surface (*F*), small pure bending, and linear material elasticity, pillar bending can be modeled by simple beam theory, see **Figure** **6**a^[^ ^17^, ^127^ ^]^

(1) $$F=\frac{3\pi EI}{L^{3}}\Delta x$$

where *E* is Young's modulus of pillar material, *L* denoted the pillar height, and *I* and Δ*x* signify the second moment of inertia of the pillar cross section and pillar top displacement, respectively. Lemmon et al. compared the practical pillar bending geometry with theoretical pillar bending patterns under pure traction force and traction force with applied moments to assess whether cells exert any moments from the focal adhesions.^[^ ^128^ ^]^ They visualized the entire pillar by coating the pillar lateral surfaces with fluorophore‐conjugated BSA and slicing with confocal microscopy. Their results suggest that the pure traction force model is accurate enough to estimate CTFs. Pillar top displacement will contain components from lateral shear^[^ ^129^ ^]^ and a tilt due to substrate wrapping, even under a pure shear force, see Figure 6b.^[^ ^129^ ^]^ Substrate shear and wrapping are more prominent in low aspect ratio pillars. Schoen et al. suggested that forces might be overestimated by up to 45% in the published literature due to the disregard for substrate wrapping.^[^ ^129^ ^]^

Additionally, in PDMS, pillars often undergo surface oxidation before protein absorption. This process forms a thin silicate layer on the PDMS surface, reaching up to 70 GPa in elastic modulus.^[^ ^130^ ^]^ This layer can drastically alter overall mechanical properties regardless of its extremely small thickness. Yong et al. studied the effect of surface oxidation on pillar mechanics and reported that oxidation could change overall stiffness by more than 200%.^[^ ^131^ ^]^ DRIE is frequently utilized to fabricate pillars or molds for high aspect ratio structures. Alternating etching and passivation steps in DRIE form a regular pattern of notches on the pillar's surface. This could lead to overestimating the effective pillar diameters, and corrections are evident in the literature for CTF calculations.^[^ ^31^ ^]^ Finite‐element analysis is employed in many instances to model the pillar bending. However, even with numerical analysis, geometrical deviations, material heterogeneities, and unidentified boundary conditions could be present.

Due to potential calculation uncertainties, researchers often employ calibration techniques for pillar characterization. Atomic force microscope (AFM) is a commonly used calibration technique.^[^ ^132^ ^]^ Alternatively, some researchers have utilized the deflection of a pulled glass micropipette to calibrate micropillars. They pre‐calibrated the micropipette using precisely weighed gold wires.^[^ ^133^ ^]^ An alternative calibration approach involves assessing pillar deflection under the weight of a p‐nitrophenol crystal, which can be precisely measured through a chemical method.^[^ ^24^ ^]^ Piezoresistive cantilevers have been employed for the calibration of relatively larger pillars.^[^ ^134^ ^]^ Also, even when the pillar's spring constant is analytically calculated, the aforementioned calibration methods are often used to estimate the material's Young's modulus. In the case of nanopillars, where the previous methods are impractical, eigenfrequencies of the pillars have been utilized to estimate Young's modulus.^[^ ^99^ ^]^

### 3.3 Micropillar Deflection Measurement

**Figure 7:**
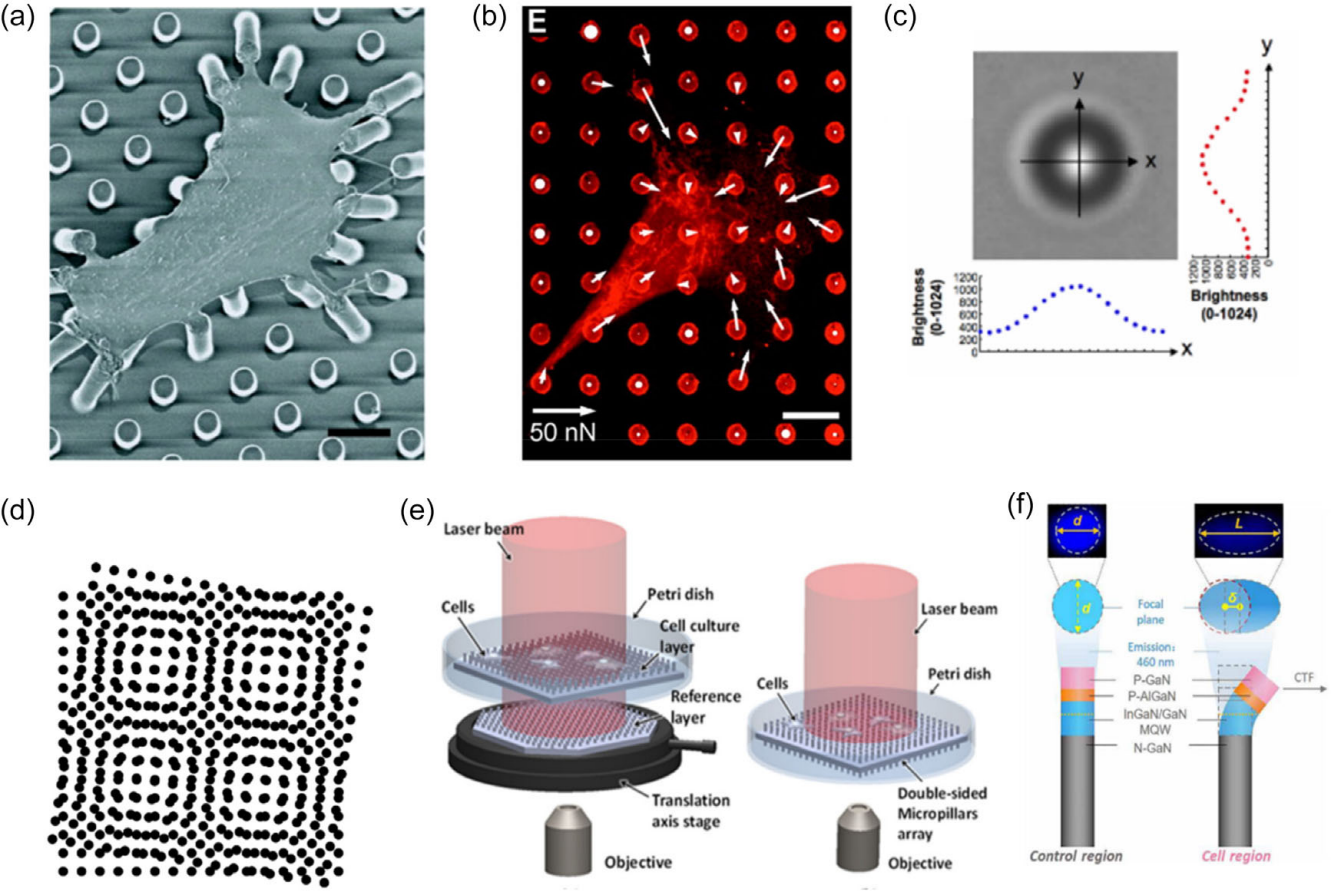
Micropillar deflection measurement techniques. a) Scanning electron microscopy (SEM) image of a smooth muscle cell attached to a micropillar array. SEM images are utilized to observe cell attachment geometries and morphologies. As live cells cannot be imaged with SEM, it is not typically used for CTF measurements. b) Immunofluorescence imaging of smooth muscle cells for CTF measurements. White arrows indicate the forces and directions on each pillar measured by top displacement. (a,b) Reproduced with permission.^[^ ^15^ ^]^ Copyright 2004, National Academy of Sciences. c) Identification of the centroid of a pillar by fitting Gaussian curves on pixel brightness in a brightfield image of a micropillar. This technique is commonly used for fluorescence images as well. Reproduced with permission.^[^ ^205^ ^]^ Copyright 2011, Elsevier. d) Example of a moiré fringe pattern created by overlaying two circular dot arrays in a square arrangement. e) Two micropillar arrays stacked vertically and a double‐sided micropillar array induce moiré fringe patterns. Reproduced with permission.^[^ ^143^ ^]^ Copyright 2014, AIP Publishing. f) Change in photoluminescence effect of InGaN/GaN piezo‐phototronic nanopillars based on strain. Reproduced under terms of CC BY‐NC 4.0.^[^ ^145^ ^]^ Copyright 2021, The Authors. Published by American Association for the Advancement of Science.

In the majority of experiments, vision‐based techniques are employed to measure the top displacements of micropillars. To enhance visualization and distinguish it from the background, pillar tops are commonly labeled with fluorescent dyes. A wide range of dyes, such as DiI (1,1′‐dioctadecyl‐3,3,3′,3′‐tetramethylindocarbocyanine perchlorate), DiD (1,1′‐dioctadecyl‐3,3,3′,3′‐tetramethylindodicarbocyanine, 4‐chlorobenzenesulfonate salt), Alexa Fluor 647, 488, 594, and various organic fluorophores, are documented in the literature. Measurements typically involve fluorescence microscopy, with image analysis conducted by comparing with an undeflected pillar pattern (**Figure** **7**b)^[^ ^14^ ^]^ or fitting an ideal grid to the array using pillars unattached to cells. Often, the edges of pillar tops are not distinctly visible, leading to the use of a 2D Gaussian curve fitted on pixel brightness for estimating pillar locations, see Figure 7c.^[^ ^19^, ^135^ ^]^

Custom applications are frequently used for tracking displacements, although some studies also employ particle tracking software like ImageJ,^[^ ^136^ ^]^ G‐Track,^[^ ^137^ ^]^ and artificial intelligence (AI)‐based object tracking systems, such as MtrackJ.^[^ ^138^ ^]^ A notable method involves comparing pillar tops with optically sliced images of pillar bottoms to eliminate global errors and displacements due to substrate lateral shear.^[^ ^132^ ^]^ Some experiments have used photoluminescent GaInP tips on GaP pillars, exhibiting stable photoluminescence and minimal photobleaching.^[^ ^139^ ^]^ Quantum dots, known for their high photostability,^[^ ^133^ ^]^ have also been embedded within microstructures to track displacements, especially in studies where pillar tops are not accessible. Unlike other methods, Qdots can be embedded within the microstructures, and researchers have used these embedded Qdots to track the displacement of micropillars where pillar tops were bonded with a PDMS cover.^[^ ^140^ ^]^ While fluorescence imaging is the predominant method for measuring pillar displacement, brightfield imaging has also been reported to provide significantly higher displacement measurement rates than fluorescence methods.^[^ ^141^ ^]^ Scanning electron microscopy (SEM) is often employed to observe cellular morphologies on micropillars (Figure 7a). Samples are coated with gold or similar noble metals to make them conductive before imaging. However, it is noted that SEM cannot be used for live CTF measurements as cells cannot survive the preparation procedure and the extreme vacuum in SEM imaging.

Zhang et al. proposed using moiré fringe patterns to enhance the visualization of pillar displacement.^[^ ^142^ ^]^ The moiré effect is created by overlaying one repetitive pattern with another similar or different repetitive pattern, see Figure 7d. Minor distortions in one pattern lead to more pronounced distortions in the final Moiré fringe pattern. Zhang et al. conducted experiments using two‐angled coherent light beams and two stacked, slightly misaligned, periodically patterned micropillar substrates.^[^ ^143^ ^]^ The same researchers later developed a double‐sided micropillar array, see Figure 7e. In this approach, forces are estimated by the distortion of the Moiré fringe pattern created by the displacement of one set of pillars. This method is well suited for high‐throughput, real‐time force mapping.

The piezo‐phototronic effect, a synthesis of piezoelectricity, semiconductor properties, and photoexcitation, was first introduced by Wang in 2010.^[^ ^144^ ^]^ To map CTF, Zheng et al. utilized the photoluminescence of multiple quantum wells at the tips of InGaN/GaN piezo‐phototronic nanopillars.^[^ ^145^ ^]^ In these pillars, photoluminescence is highly sensitive to piezo potential. Forces applied to the pillars alter the intrinsic strains, leading to a redistribution of piezo potential. Changes in piezo potential regulate photoluminescence, which becomes observable under excitation. This methodology allows for the simultaneous use of pillar top displacement and changes in photoluminescence intensity to estimate forces, see Figure 7f.

The precision and resolution of micropillar deflection measurement directly affect the accuracy of CTF estimation. By tuning the micropillar stiffness, the level of pillar deflection can be adjusted to suit the specific application. Cellular forces vary widely depending on the type of force being measured: subcellular forces can be in the picoNewton (pN) range, while tissue‐level forces can reach millinewton (mN) levels.^[^ ^23^ ^]^ The smallest force measurement evident with micropillars was 15 pN, where researchers measured forces exerted by subcellular activities on a gallium phosphide nanopillar array with a stiffness of 0.312 nN μm^−1^.^[^ ^99^ ^]^ Commonly, CTF measurements with micropillars range from 0.1 to 100 nN, with micropillar stiffness values ranging from 1 to 100 nN μm^−1^.^[^ ^15^, ^18^, ^23^, ^125^, ^146^, ^147^ ^]^ However, in experiments with highly contractile cells like cardiomyocytes, forces up to 400 nN on micropillars have been observed in the literature.^[^ ^148^ ^]^ The micropillar technique does not have a strict upper limit for force measurement. For instance, skeletal muscle myoblast tissues grown between micropillars in 3D have demonstrated forces reaching up to 800 mN.^[^ ^149^ ^]^

### 3.4 Applications in Cellular Forces Sensing

#### 3.4.1 Focal Adhesion Dynamics

Focal adhesions are the force‐transferring and mechanosensing component of cells, so studying the dynamic behavior of focal adhesion and focal adhesion proteins has become an attractive application of micropillars.^[^ ^150^ ^]^ Heli et al. explored the growth of focal adhesions under lateral shear forces exerted on fibroblasts via micropillars, correlating the exerted force with focal adhesion length, growth rates, and paxillin intensity.^[^ ^151^ ^]^ Similarly, another research group utilized a micropillar substrate coated with gold nanoparticles to study the contribution of *α*_v_ *β*_3_ and *α*_5_ *β*_1_ on CTFs by selectively inhabiting each integrin protein.^[^ ^42^ ^]^ Hoorn et al. enhanced the focal adhesion study approach by integrating the conventional fluorescence imaging of micropillar displacement with super‐resolution microscopy to study the nanoscale architecture of focal adhesion complexes.^[^ ^152^ ^]^ This allowed the team to correlate molecular arrangements with the mechanical forces exerted. The setup facilitated observation from both sides simultaneously by covering the substrate with a thin coverslip, using microspacers on the pillar array in an inverted configuration.

Cadherin‐mediated cell–cell adhesions also exist in multicellular assemblies, in addition to focal adhesions with ECM. Thus, focal adhesion dynamics not only depends on individual cell mechanobiology but also signals from neighboring cells.^[^ ^153^ ^]^ Micropillar‐based studies of multicellular assemblies have shown that force transmission to ECM through focal adhesion is achieved as a collective behavior of cells and forces are concentrated on edges of cell assemblies similar to single cells.^[^ ^33^ ^]^ Studies on morphogenesis have shown that cell–cell adherence force positively correlates with focal adhesion forces.^[^ ^154^ ^]^ Broussard et al. utilized a micropillar array to study the role of desmosome–intermediate filament complexes, which play a crucial role in cell adhesion.^[^ ^155^ ^]^ They show that strengthening desmosome–intermediate filament results in an increase in both focal adhesion and cell–cell adhesion forces.

#### 3.4.2 Cardiomyocytes Contraction

The heart's pumping action primarily relies on the contraction of cardiomyocytes, and dysfunctions in these cells are significant contributors to cardiac diseases. Cardiomyocytes intrinsically generate force, making contractile force measurement a direct method to assess their functionality.^[^ ^156^ ^]^ The use of micropillar arrays for analyzing both cardiomyocytes and stem‐cell‐derived cardiomyocytes is well documented in several studies.^[^ ^157^, ^158^ ^]^ These studies involve 2D cell cultures on micropillar arrays and focus on measuring the tangential traction forces exerted by the cells.^[^ ^145^, ^156^ ^]^ Micropillar platforms allow for the study of cardiomyocytes’ time‐dependent contraction forces (beating).

**Figure 8:**
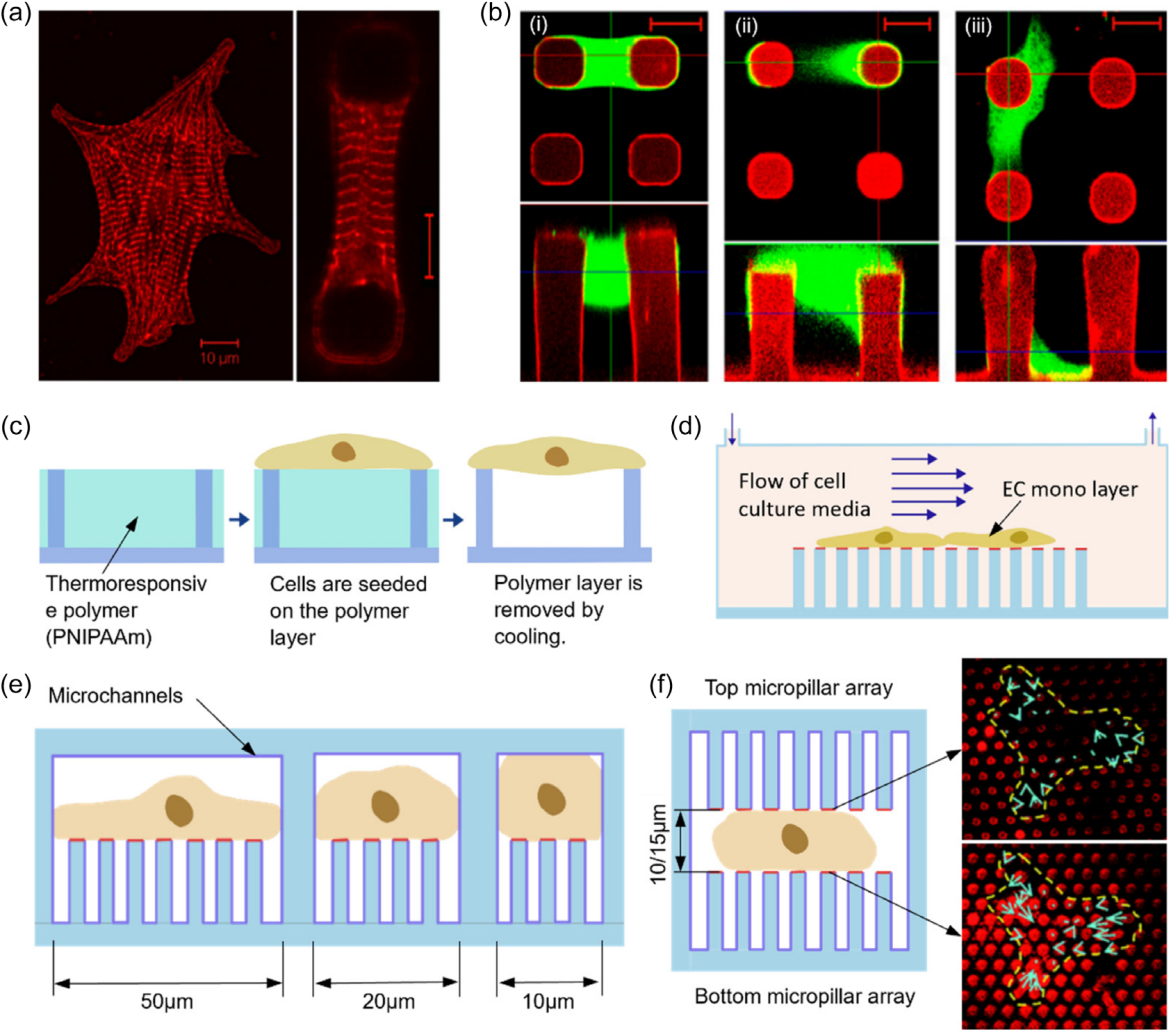
Micropillar‐based cellular force measurement. a) Comparison of 2D cardiomyocyte growth on a flat surface versus suspension between two micropillars in 3D, demonstrating a regular Z‐band pattern in 3D morphology. b) Practical geometries of 3D cell attachment between two micropillars: i) 1st attachment is ideal, and ii) forces can be estimated with 2nd attachment, but calculations become complex, and iii) 3rd attachment is undesirable. (a,b) Adapted with permission.^[^ ^148^ ^]^ Copyright 2008, Elsevier. c) Use of a thermoresponsive polymer layer to limit cell attachment exclusively to the tops of pillars, preventing undesirable 3D attachment patterns. Reproduced with permission.^[^ ^102^ ^]^ Copyright 2020, Springer Nature. d) Examination of shear force‐induced changes of CTFs and cell morphology in endothelial cell monolayers cultured within a microfluidic flow chamber, where culture media is circulated. Reproduced with permission.^[^ ^162^ ^]^ Copyright 2012, Royal Society of Chemistry. e) Measurement of CTFs as cells migrate within confined microchannels. The internal surfaces of these channels are coated with pluronic to restrict force generation solely to interactions with the pillar tops. Reproduced with permission.^[^ ^138^ ^]^ Copyright 2013, Royal Society of Chemistry. f) Double‐sided micropillar array to mimic a 3D environment for cell migration. Adjusting the distance between arrays controls cell confinement. Right‐side images show force vectors on top and bottom arrays for a 10 μm gap, with magnitudes of forces being larger on the bottom array than the top one. Reproduced with permission.^[^ ^177^ ^]^ Copyright 2019, Royal Society of Chemistry.

Since cardiomyocytes are organized in 3D structures in vivo, studies have shown significant differences in cell morphology, contractile ability, adhesion structures, and few other aspects between 2D and 3D organized cardiomyocytes.^[^ ^159^ ^]^ Kajzar et al. adjusted the separation distance between micropillars to approximately match the size of myocytes, enabling the cells to suspend between two pillars.^[^ ^148^ ^]^ This arrangement led to observations of more compact, spindle‐like cell morphologies with sarcomeric myofibers extending across the cell and regular Z‐band patterns, closely mimicking in vivo conditions, see **Figure** **8**a. However, this experiment did not confine cell attachment to the pillar tops (Figure 8b), resulting in uncertain cell attachment geometries. To overcome this, Taylor et al. filled the pillar array with a thermoresponsive polymer prior to seeding the cells.^[^ ^134^ ^]^ After the cells were attached, the sacrificial layer was dissolved by cooling the media, see Figure 8c. This approach not only achieves a well‐defined force geometry but also preserves the advantages of 3D cell morphology.

#### 3.4.3 Hemodynamic and Trans‐Endothelial‐Flow‐Associated Forces

Endothelial cells line the interior layer of blood vessels and, in vivo, are continuously subjected to forces from hemodynamics and trans‐endothelial flow.^[^ ^160^ ^]^ Research has shown that endothelial cells are highly sensitive to shear stresses, and abnormalities in these cells can lead to several vascular diseases.^[^ ^161^ ^]^ To explore the flow‐mediated mechanotransduction properties of endothelial cells, Raymond et al. developed a micropillar array within an enclosed fluid channel to study the subcellular forces exerted on human umbilical vein endothelial cells under fluid shear forces, see Figure 8d.^[^ ^162^ ^]^ Their investigation focused on flow‐induced cell elongation and cytoskeleton contractions in these cells.

Trans‐endothelial migration is the process by which cells migrate through the endothelial cell barrier. This process is particularly critical in the immune response, especially the trans‐endothelial migration of leukocytes, which includes several steps: capturing, rolling, firm adhesion, intravascular crawling, and transmigration.^[^ ^163^ ^]^ To assess the mechanical forces associated with these processes, endothelial cell monolayers were seeded on micropillar arrays, and forces were monitored as leukocytes transmigrated.^[^ ^164^ ^]^ These experiments have documented radially outward traction forces around the point of transmigration. In a related study, Zhijun et al. reported that, even before transmigration begins, alterations in the endothelial‐cell‐generated traction forces occur during firm adhesion and early transmigration steps.^[^ ^165^ ^]^

While endothelial cells form the inner lining of blood vessels, vascular smooth muscle cells encase the endothelium and play critical roles in the contraction and relaxation of blood vessels. Micropillar arrays have been a valuable tool for exploring the intrinsic contraction properties of smooth muscle cells^[^ ^132^ ^]^ and for understanding the relationship between the morphology of focal adhesions and the forces generated by them.^[^ ^137^ ^]^ In addition to the forces produced by vascular smooth muscle cells, blood vessels also endure cyclic forces resulting from blood flow. Nagayama et al. investigated the dynamic changes of CTFs at focal adhesions by culturing vascular smooth muscle cells on a stretchable micropillar platform and applying cyclic stresses.^[^ ^125^ ^]^

#### 3.4.4 Cell Migration

Cell migration is crucial for various biological functions, including morphogenesis, immune response, tumor metastasis, and wound healing. It is inherently linked with CTFs and cell mechanosensing.^[^ ^153^ ^]^ The use of micropillars to study the migration of various cell types such as fibroblasts,^[^ ^166^, ^167^ ^]^ epithelial cells,^[^ ^168^ ^]^ dendrites,^[^ ^169^ ^]^ and cancer cells^[^ ^170^ ^]^ has been extensively documented in the literature.

Micropillars featuring areas of differing stiffness, induced by micropillar heights^[^ ^30^ ^]^ and stepped micropillars with varying base diameters,^[^ ^171^ ^]^ have been employed to investigate the impact of substrate rigidity on cell migration. These studies indicate that cells tend to migrate toward stiffer regions,^[^ ^34^ ^]^ and the magnitudes of CTFs positively correlate with pillar stiffness.^[^ ^124^ ^]^ This directional migration pattern is known as durotaxis.^[^ ^172^ ^]^ However, in most of these studies, stiffness changes in discrete steps, meaning only cells at boundary regions can sense the stiffness difference and, thus, cells are not exposed to a true stiffness gradient. Research involving pillar arrays with anisotropic pillar rigidities also shows that cells tend to migrate along the direction of greater stiffness.^[^ ^168^ ^]^

In addition to pillar stiffness, recent studies have suggested that the micropillar arrangement‐induced ECM density influences cell migration.^[^ ^173^ ^]^ This phenomenon, termed topotaxis, has been explored using micropillar arrays with gradients in pillar density.^[^ ^170^ ^]^ However, unlike durotaxis, topotaxis exhibits complex cellular migration patterns that depend on the cell type and biochemical cues.^[^ ^174^ ^]^

Due to their extendable sensing area, micropillar arrays are an effective platform for studying multicellular assemblies. Saez et al. investigated the collective migration mechanics of multicellular assemblies of Madin–Darby canine kidney (MDCK) epithelial cells on micropillar arrays.^[^ ^33^ ^]^ They observed that CTFs strongly depend on the cell's relative position within the assembly. The largest pillar deflections were noted at the edges of the cell islands, while internal forces demonstrated that force balance is achieved through collective behavior.

In vivo, cell migration occurs through a 3D matrix, and cells exhibit a variety of migratory mechanisms in this environment. Interestingly, even a single cell can switch migration mechanisms in response to changes in the external environment.^[^ ^175^ ^]^ Researchers have developed both bead‐embedded continuous gel substrates^[^ ^176^ ^]^ and micropillar devices^[^ ^126^ ^]^ to measure CTFs while simulating confined 3D cell migration. Raman et al. fabricated micropillar arrays inside microchannels with widths of 10, 20, and 50 μm, see Figure 8e.^[^ ^138^ ^]^ They induced the migration of fibroblasts and human osteosarcoma cells through chemotaxis and found that CTFs were reduced in narrower channels. Further, they treated cells with blebbistatin and calyculin A, significantly reducing and increasing CTFs in 2D cell migration. However, in confined channels, they reported no changes in CTFs or migration speed with these treatments, suggesting adhesion‐independent migration modes.

Hui et al. advanced this research by experimenting with a more developed version of a confined CTF measurement system.^[^ ^177^ ^]^ They fabricated micropillar arrays on opposing sides of two parallel PDMS substrates, with osteoblastic cells seeded between the pillar arrays, see Figure 8f. CTFs were measured for two different substrate separation gaps, revealing reduced CTFs and higher migration rates in narrower gaps.

#### 3.4.5 Morphogenesis

**Figure 9:**
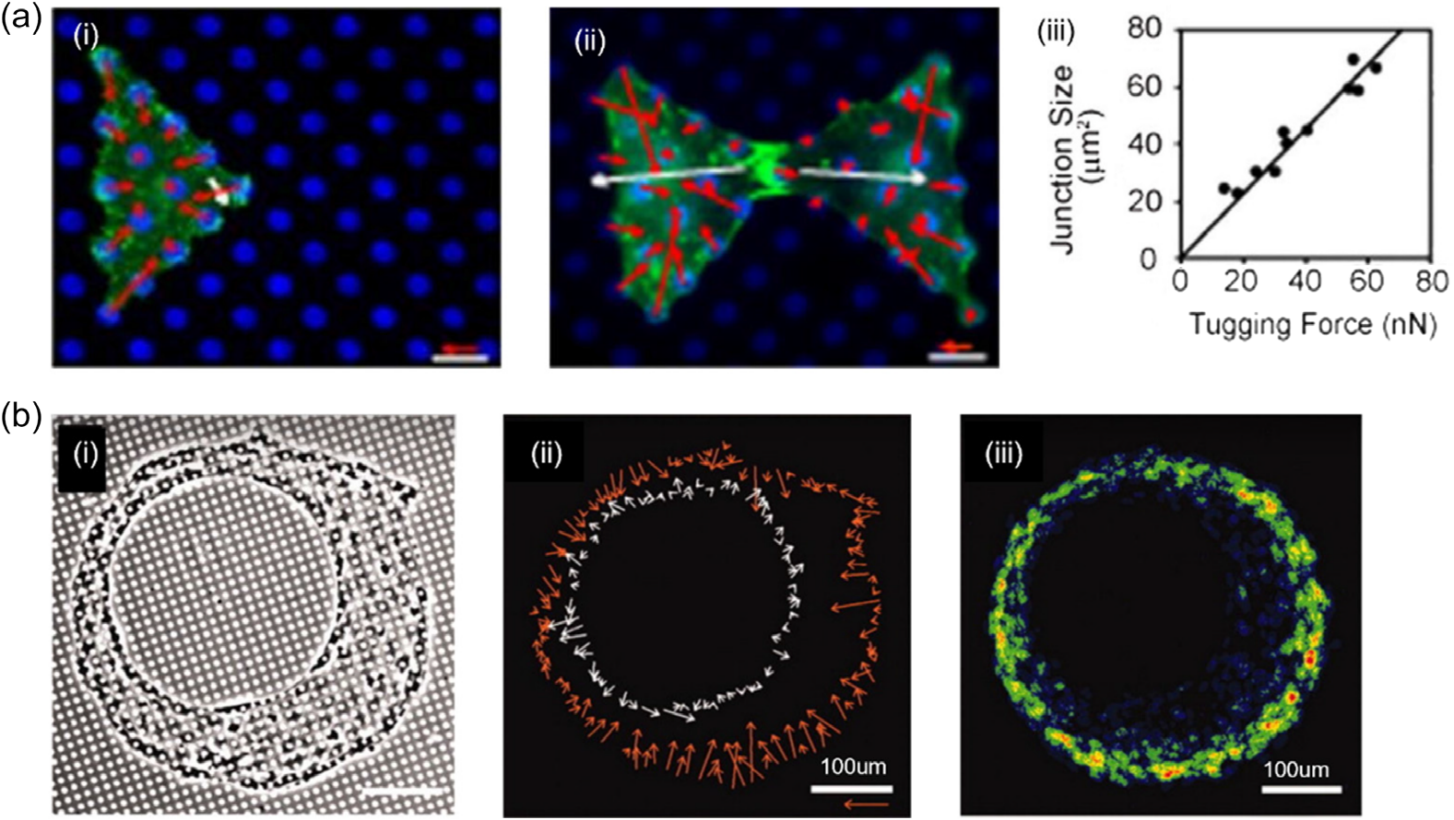
Traction forces in multicellular assemblies. a) Measurement of tugging force between two cells at an adherence junction. i, ii) Force balance within a single cell and two cells printed in the bowtie pattern. iii) Graph shows the correlation between adherence junction size and tugging force, with each point representing a pair of cells. Reproduced with permission.^[^ ^154^ ^]^ Copyright 2010, National Academy of Sciences. b) i) Epithelial cell monolayer patterned into excentric annular shape on micropillar array. ii) The second figure shows the spatial distribution of force vectors along the edge of the annular shape, and iii) the color metric map indicates the cell proliferation rate across the monolayer. Reproduced with permission.^[^ ^187^ ^]^ Copyright 2005, National Academy of Sciences.

Morphogenesis is the underlying process that causes multicellular organisms to take shape,^[^ ^178^ ^]^ with mechanical forces playing a critical role.^[^ ^179^ ^]^ In multicellular assemblies, cell–cell adherence junctions mediate force transfer between cells. Using a micropillar array, Liu et al. studied the tugging force generated at adherence junctions between cells.^[^ ^154^ ^]^ They patterned two epithelial cells in the shape of a bow tie on micropillars, positioning the adherence junction at the narrowest point, see **Figure** **9**a. They analyzed the relationship between adherence junction size and tugging force through myosin activation and inhibition. Additionally, they investigated the growth of adherence junctions under externally induced tugging forces. Their findings reveal a linear positive relationship between cell–cell tugging force and adherence junction size.

Wesley et al. developed a micropillar platform to investigate the forces generated by microtissues while remodeling in 3D space.^[^ ^180^ ^]^ The pillars were fabricated in pairs and placed in collagen‐filled wells to restrict tissue attachment to two pillars. They used multilayer photolithography to fabricate wider pillar heads, ensuring tissue anchoring on the pillars even after large deflections. Their findings suggest that multiple mechanical inputs, including boundary mechanics, pillar stiffness, and mechanical stresses, affect the forces exerted by the tissue. Further developments included micropillar pair platforms with an integrated micronickel sphere on one pillar of each pair to induce an external force via a magnetic field.^[^ ^181^, ^182^ ^]^ Microtissue growth and the forces generated on multi‐pillar units with more than two pillars have also been experimentally studied.^[^ ^36^, ^140^, ^183^ ^]^ Jaemin et al. studied and modeled the response to a surgical cut on a microtissue using a similar platform with four anchoring micropillars in a well.^[^ ^184^ ^]^

#### 3.4.6 Cell Proliferation

Cell proliferation is the process of cell multiplication, regulated by both intracellular gene regulatory networks and extracellular factors.^[^ ^185^ ^]^ This includes both cell division and cell growth. Ogura et al. discussed mechanical stresses as a mediating factor in cell proliferation.^[^ ^186^ ^]^ To explore this phenomenon, Nelson et al. patterned epithelial cell monolayers in an annular shape on micropillars.^[^ ^187^ ^]^ This configuration induces higher cell traction stresses on the outer convex edge than on the inner concave edge. Their results demonstrate a robust positive correlation between cell proliferation rates and cell traction stresses, see Figure 9b.

Articular cartilage is a specialized type of tissue that provides lubrication and load‐bearing between bones. Chondrocytes, the cells responsible for forming articular cartilage, are naturally arranged in a complex, layered orientation that is difficult to mimic artificially. Janssen et al. utilized a vertically aligned multi‐wall carbon nanotube array to control chondrocyte proliferation.^[^ ^108^ ^]^ The high surface area and chemical absorption ability of multi‐wall carbon nanotubes help to structurally mimic collagen fibers. The nanosurface structure of the tube array provides strong anchorage points to cells. The research group could control proliferation by optimizing individual pillar and pillar arrangement parameters, achieving unidirectional chondrocyte growth. Furthermore, they compared the results with similar Si/SiO_2_ geometries, demonstrating that vertically aligned multi‐wall carbon nanotubes have superior ECM‐imitating abilities.

#### 3.4.7 Drug Screening

Although conventional microfluidic devices are widely used in drug screening,^[^ ^188^, ^189^, ^190^ ^]^ the application of micropillar or micropillar‐like platforms has been explored in only a few studies.^[^ ^191^ ^]^ Herman et al. developed a micropillar platform to assess the impact of specific drugs on the strength of myofibers grown between two pillars.^[^ ^149^ ^]^ They utilized two platinum electrodes placed in the culture media to achieve maximum tetanic force through electrical stimulation. Similar to CTF measurements, pillar deflection was used to compare the forces before and after drug treatments. In another study, Li et al. employed a nanopillar array to explore the effect of α‐difluoromethylornithine, an anticancer drug, on MCF7 breast cancer cells and MCF10A non‐cancer cells.^[^ ^139^ ^]^ They observed notably high CTFs in cancer cells prior to treatment and a significant decrease in CTFs in cancer cells posttreatment but not in healthy cells. Furthermore, they discussed the potential of CTFs as a cancer biomarker.

Muscle strength is critical to animal locomotion and can decline due to diseases and aging. Nemaflex is a micropillar‐based platform developed to measure muscle strength. This platform uses an inverted micropillar array to record pillar deformations caused by crawling *Caenorhabditis elegans*, providing insights into muscle strength.^[^ ^192^ ^]^ Researchers have used this device to study the effects of various drugs on muscle strength. Additionally, the Nemaflex platform was later utilized to investigate the effects of microgravity on muscle strength aboard the International Space Station.

### 3.5 Nanopillar Arrays

Numerous studies have employed nanopillar arrays to measure cellular forces down to the picoNewton (pN) level.^[^ ^99^, ^100^, ^193^, ^194^ ^]^ The exceptional spatial resolution and sensitivity of these nanopillars enable accurate measurements of forces associated with subcellular activities, such as the formation of filopodia and lamellipodia. Cell motility begins with the extrusion of actin‐rich cell membranes, known as filopodia,^[^ ^195^ ^]^ which can detect topological features as small as 8 nm^[^ ^196^ ^]^ and generate retraction forces at the pN level through adhesions.^[^ ^197^ ^]^ Understanding the forces generated by these structures, especially filopodia, is crucial for grasping the underlying principles of mechanosensing. Moreover, experiments have indicated that unusually high filopodia activity, a characteristic of cancer cells, contributes to their enhanced motility.^[^ ^198^ ^]^ Hallstrom et al. developed a 40 nm thick gallium phosphide pillar array on silicon to measure forces exerted by neural growth cones.^[^ ^99^ ^]^ These pillars, grown using metal‐organic vapor phase epitaxy, could measure forces as small as 15 pN.

## 4 Micropillar Arrays with Actuation

Mechanotransduction involves converting mechanical stimulations into cellular responses, including contractile activations, adhesion regulations, and intracellular signaling.^[^ ^199^ ^]^ Micropillar platforms with actuation capabilities provide a powerful method to deliver mechanical stimulation and sense mechanical response simultaneously.

### 4.1 Magnetic Actuation

**Figure 10:**
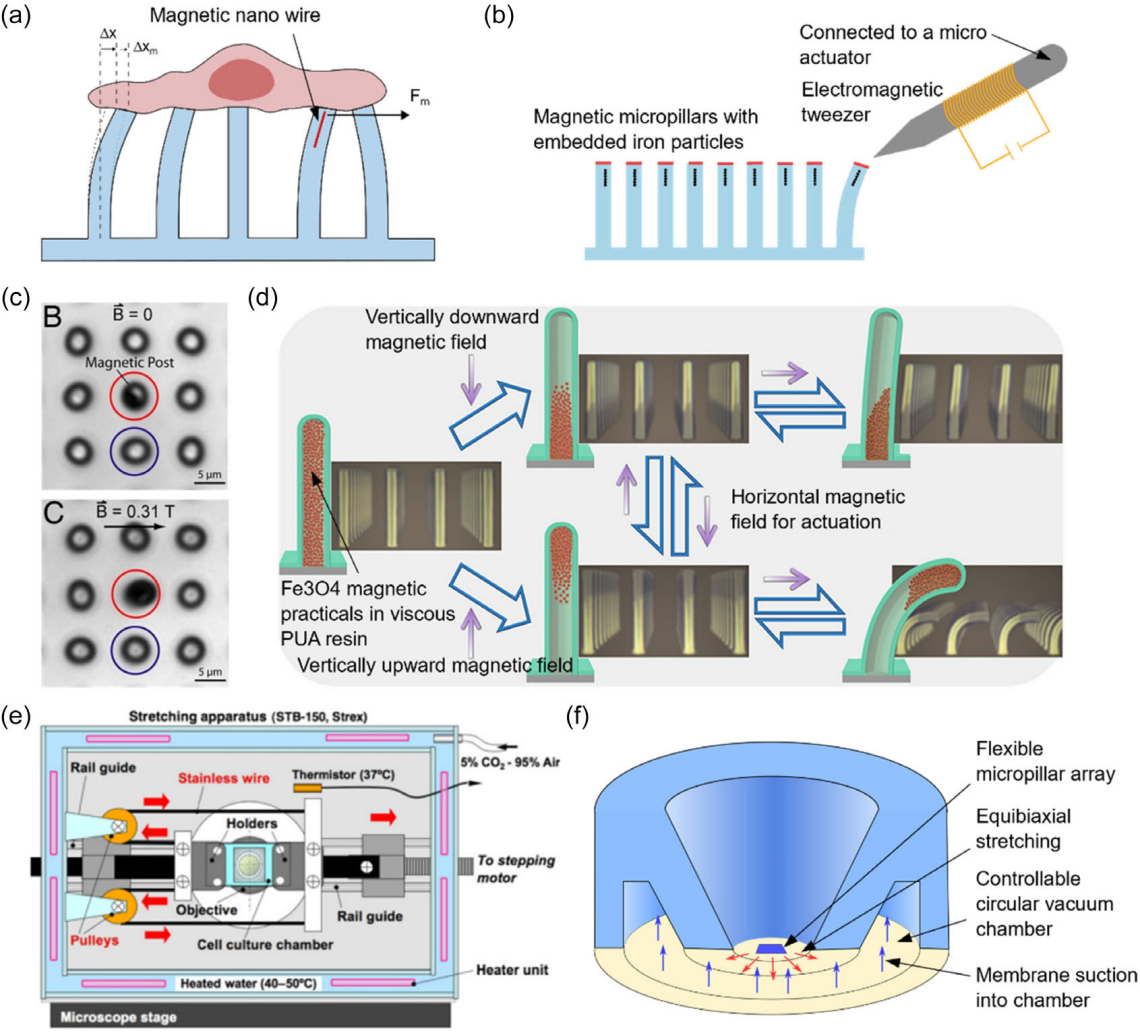
Micropillar actuation methods. a) Basic geometry of mechanostimulation via micropillar‐embedded nanowire. The magnetic dipole moment of the wire generates an unbalanced force (*F*_m_) on the micropillar. This stimulation alters the deflections of other pillars through the mechanotransduction process (Δx_m_). Reproduced with permission.^[^ ^166^ ^]^ Copyright 2007, National Academy of Sciences. b) Induction of local magnetic force by magnetic tweezer. Reproduced with permission.^[^ ^202^ ^]^ Copyright 2018, Springer Nature. c) Deflection of a magnetic pillar under a horizontal magnetic field. The marked pillar is deflected about 800 nm under a 0.31T magnetic field. Reproduced with permission.^[^ ^201^ ^]^ Copyright 2007, National Academy of Sciences. d) Programmable magnetic micropillars. By exerting a vertical magnetic field, magnetic particles are relocated to the top or bottom position within the micropillar, affecting pillar deflection based on the vertical position of magnetic particles. Reproduced with permission.^[^ ^203^ ^]^ Copyright 2021, American Chemical Society. e) Uniaxial stretching of micropillars on a modified STREX cell platform. Reproduced with permission.^[^ ^205^ ^]^ Copyright 2011, Elsevier. f) Equibiaxial stretching of a micropillar array. By controlling the vacuum, the stretching of the micropillar array is regulated. Reproduced with permission.^[^ ^206^ ^]^ Copyright 2012, Oxford University Press.

Magnetic actuation is favored for micropillar manipulation due to its remote‐control capability, lack of interaction with biological or chemical substances, immediate response, and high selectivity.^[^ ^200^ ^]^ Integration of cobalt nanowires in PDMS micropillars allows the pillars to bend with an external magnetic field, a technique explored in research, see **Figure** **10**a.^[^ ^141^ ^]^ Nanowires, suspended in ethanol, are vertically aligned at the top of the pillars using a vertical magnetic field during the PDMS casting stage. A horizontal magnetic field is applied for actuation, causing the pillars to bend due to the magnetic dipole moment on the nanowires, see Figure 10c.^[^ ^201^ ^]^ Prior to experimentation, the locations of magnetic micropillars were mapped and characterized for deflection induced by the magnetic field.^[^ ^135^ ^]^ Initially, the placement of magnetic pillars was uncontrolled. Later, Nagayama et al. employed a patterned PDMS mask to control the deposition pattern of magnetic particles during casting.^[^ ^146^ ^]^ In this experiment, microiron particles served as the magnetic material, achieving an average force of 162 nN, compared to 45 nN with nanowires. Later, they developed an electromagnetic tweezer to apply a localized magnetic field, observing smooth muscle cells under local cyclic stretches, see Figure 10b.^[^ ^202^ ^]^

Ni et al. developed a micropillar array capable of programmable actuation by first fabricating a hollow micropillar array with polyurethane acrylate using a positive pillar array die on a microhole mold filled with resin.^[^ ^203^ ^]^ The micropillar shells were then filled with superparamagnetic iron oxide particles coated with silica shells in an uncurable, highly viscous polyurethane acrylate resin and sealed at the bottom with a polyethylene terephthalate backing layer. The position of the paramagnetic particles along the pillar axis could be altered with a vertical magnetic field, affecting the amount of pillar bending under a horizontal magnetic field, see Figure 10d. Most recent advancements in magnetically responsive micropillars, their fabrication techniques, and applications are discussed elsewhere.^[^ ^204^ ^]^

### 4.2 Substrate Stretching

In vivo vascular endothelial cells and smooth muscle cells are constantly subjected to microscopic stretching due to hemodynamic and external forces. Stretching micropillar arrays while culturing cells on them provides a platform to study whole‐cell stretching‐induced CTF changes.^[^ ^205^ ^]^ Nagayama et al. experimented with a micropillar‐based cell stretching platform using a commercially available cell stretching platform (STREX cell), modified for symmetrical stretching to maintain the area of interest within the field of view, see Figure 10e.^[^ ^125^ ^]^ They observed CTF changes with cyclic uniaxial stretching of the substrate up to 6%, reporting that one group of smooth muscle cells showed synchronous contraction with stretching while another maintained constant CTFs. Lam et al. developed a similar platform with equibiaxial stretching capability, bonding a micropillar array on a PDMS membrane stretched by drawing the periphery to a circular vacuum chamber around the array, see Figure 10f. This method achieved a controlled equibiaxial stretch of up to 18%.^[^ ^206^ ^]^

### 4.3 Other Actuation Techniques

Photothermal and photochemical actuation of micropillars have been explored in recent studies.^[^ ^28^ ^]^ Photothermal actuation, based on the heat generation within the material through light absorption, allows precise control of the actuation pattern by manipulating light's exposed area and direction. SunBOT is a micropillar system capable of tracking the direction of white light. A wide range of soft materials, including thermoresponsive hydrogels, can be utilized for photothermally actuated micropillars.^[^ ^207^ ^]^ In photochemical actuation, chemical changes within the material, rather than the heat generation, cause mechanical deformations. Azobenzene is a molecule that undergoes cis‐trans photoisomerization upon absorbing UV light, is associated with molecular‐level contraction, and is reversible by exposure to visible light or heat.^[^ ^208^ ^]^ Polymers with azobenzene groups are commonly used in photochemical‐responsive micropillar fabrication, with pillar actuation controlled by light intensity, exposed area, and direction similar to photothermal techniques.^[^ ^209^ ^]^

Other methods, such as fluid surface‐tension‐induced actuation, shape memory alloys, and thermal actuation of liquid crystal networks, have been explored for micropillar actuation.^[^ ^28^ ^]^ While complex actuation patterns can be achieved, the literature has not explored using these techniques, including photothermal and chemical actuation, in biosensing‐related micropillar applications.

## 5 Micropillar Arrays as an Extracellular Environment

Mechanical and geometrical properties of the extracellular environment, such as rigidity, topography, and confinement, influence several cellular functions, including cell proliferation, migration, growth, and stem cell differentiation.^[^ ^197^, ^210^, ^211^, ^212^, ^213^ ^]^ Previous sections have discussed ECM mechanical‐property‐mediated migration and proliferation. This section will focus primarily on the impact of micropillar mechanical properties on cell growth, CTFs, stem cell differentiation, and cell contact guidance.

### 5.1 ECM Rigidity Modulation

Cells sense the rigidity of the ECM by exerting retraction forces through focal adhesions.^[^ ^20^ ^]^ Micropillar arrays are an effective tool for studying the effects of substrate stiffness, as pillar rigidity can be independently adjusted by altering their height without impacting the adhesion site geometry or nanoscale rigidity.^[^ ^19^ ^]^ Janssen et al. used vertically aligned multi‐wall carbon nanotube arrays to closely mimic the natural ECM rigidity of cartilage for chondrocyte growth, observing cell morphologies closer to in vivo conditions, which is difficult to achieve with conventional methods.^[^ ^108^ ^]^

**Figure 11:**
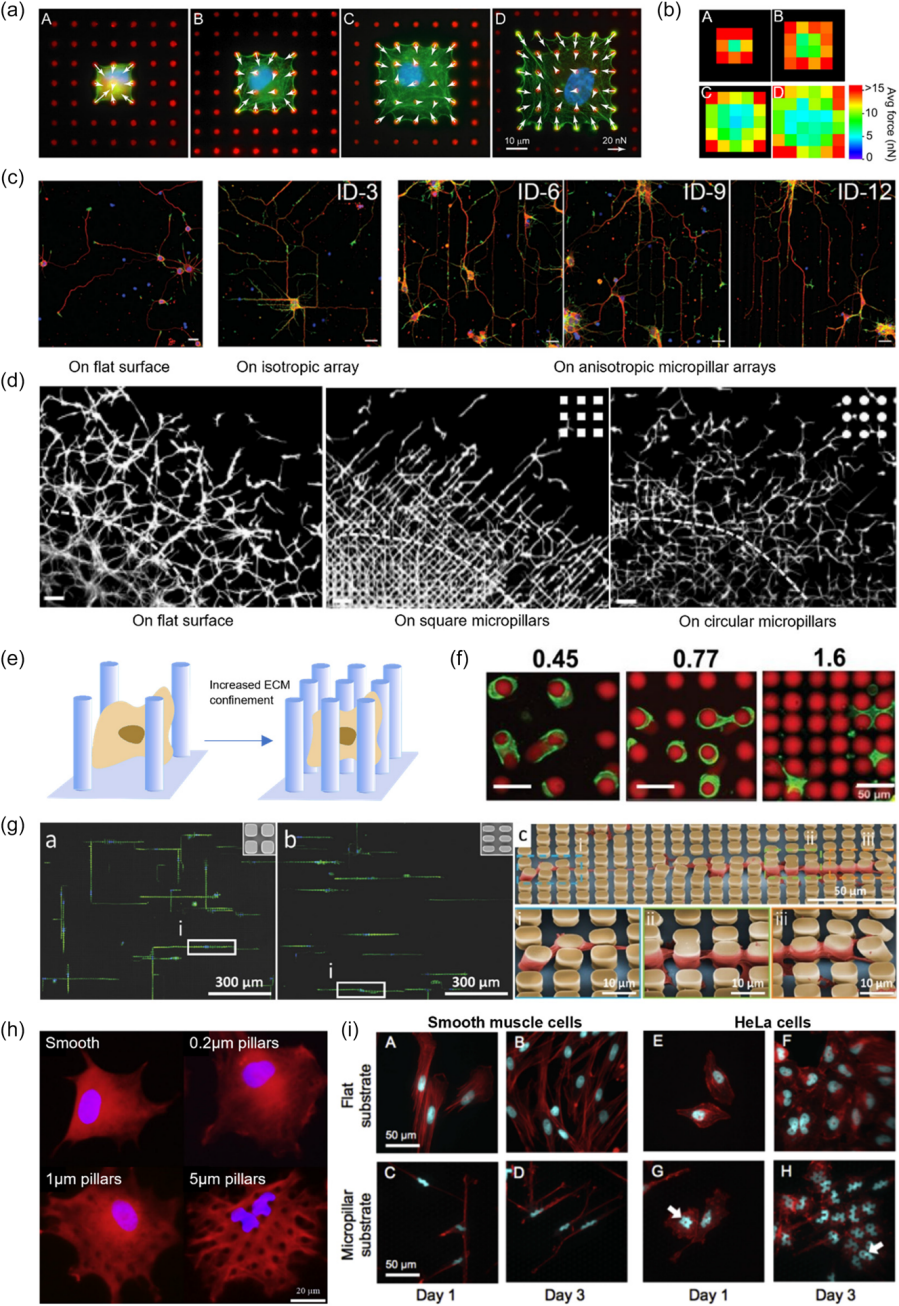
Micropillar application in ECM modulation. a) Control of cell spread area by managing fibronectin printing. This technique restricts cells to predefined areas on the micropillars. b) Color metric map displaying average traction forces on each pillar under the confined cells shown in (a). Across all configurations, high traction forces are observed at the cell's outer edges, with force concentrations at corners. However, the average force on the pillar decreases as the area increases. (a,b) Reproduced with permission.^[^ ^215^ ^]^ Copyright 2012, Elsevier. c) Neurite contact guidance on anisotropic micropillars. ID refers to the interpillar distance in the *x* direction, while the interpillar distance in the *y* direction remains constant at 3 μm. ID‐3 represents an isotropic square pillar array, whereas ID‐6, 9, and 12 exhibit increasing anisotropy. Reproduced with permission.^[^ ^229^ ^]^ Copyright 2015, John Wiley and Sons. d) Impact of individual pillar topology on cell contact guidance. Interneurons on square pillars demonstrate clear cell orientation along diagonal directions, in contrast to circular pillars of similar dimensions. Reproduced with permission.^[^ ^230^ ^]^ Copyright 2019, Elsevier. e) Modulation of 3D ECM stiffness and confinement through variations in pillar density. f) Cell morphology in different pillar densities. The numbers 0.45, 0.77, and 1.6 refer to pillar densities measured in × 10^3 ^pillars mm^−2^. (e,f) Reproduced with permission.^[^ ^238^ ^]^ Copyright 2020, John Wiley and Sons. g) Cell guidance in anisotropic 3D environments. “a” depicts an isotropic square pillar array, and “b” shows anisotropic rectangular pillar arrays. The pillar topology is illustrated in the top right corner of each figure. Cells exhibit clear guidance along the rigid axis of the rectangular pillars. “c” depicts the 3D cell morphology within the pillar array. Reproduced with permission.^[^ ^239^ ^]^ Copyright 2016, John Wiley and Sons. h) Change of bone marrow stromal cell nuclei shape with micropillar height. Reproduced with permission.^[^ ^258^ ^]^ Copyright 2012, Elsevier. i) Proliferation of smooth muscle cells and HeLa cancer cells on a flat surface and micropillar substrate. The proliferation of smooth muscle cells was significantly inhibited on micropillar substrate compared to HeLa cells. Reproduced with permission.^[^ ^269^ ^]^ Copyright 2015, Elsevier.

Califano and Reinhart‐King showed a strong positive correlation between substrate rigidity and CTFs using cells cultured on micropillar arrays.^[^ ^214^ ^]^ However, rigidity in micropillar substrates comprises components from both the individual pillar rigidity and pillar density. Han et al. conducted a comprehensive study to assess the individual effects of substrate stiffness, cell spread area, and pillar density on CTFs and focal adhesions, using human pulmonary artery endothelial cells across arrays varying in pillar stiffness and density.^[^ ^215^ ^]^ They found that both average force and spread area increased with pillar stiffness. To isolate the effect of stiffness on the spread area, they confined cells by controlling the FN printing area on the micropillars, see **Figure** **11**a,b. Their findings indicate a decrease in average force per pillar with an increase in spread area and an overall increase in total force. Moreover, with increased pillar density, cells with similar spread areas exhibited larger total forces, although the average force per pillar was lower. They also noted that the focal adhesion area reflects the average force exerted on each pillar.

Additionally, anisotropic pillar rigidity has been highlighted as a factor influencing cell morphology. Saez et al. investigated epithelial cell growth on a micropillar array with an elliptical cross section, finding that cells tend to elongate along the more rigid axis of the micropillars.^[^ ^168^ ^]^

#### 5.1.1 Stem Cell Differentiation

Stem cell differentiation is a tightly regulated process that is critical for development and homeostasis. In addition to biochemical signals, mechanobiological pathways, including ECM stiffness and microtopology, play significant roles in stem cell differentiation.^[^ ^216^, ^217^ ^]^ The modulation of stiffness using micropillar arrays has frequently been employed to study stem cell differentiation. Fu et al. explored the differentiation of human mesenchymal stem cells on micropillar arrays with varying rigidities.^[^ ^19^ ^]^ They observed that mesenchymal stem cell differentiation into osteogenic lineages was more pronounced on stiffer micropillars, while adipogenic differentiation was more prominent on softer pillars. Micropillars have also been used to control geometrical properties such as stem cell confinement through ID,^[^ ^218^ ^]^ deformation of cell nuclei,^[^ ^219^ ^]^ and variations in micropillar topology,^[^ ^220^ ^]^ all of which influence stem cell differentiation behavior. Sjöström et al. fabricated nanopillar‐like structures on titanium implants, reporting the potential for enhancing mesenchymal stem cell differentiation into osteogenic cells, which is a beneficial outcome for bone implants.^[^ ^221^ ^]^ Long et al. comprehensively reviewed the latest advancements and the effects of various micropillar parameters on stem cell differentiation.^[^ ^27^ ^]^

### 5.2 Topography

Both the topography of individual pillars and their arrangement significantly influence cellular functions.^[^ ^222^ ^]^ Reducing pillar diameter enhances the sensitivity and spatial resolution of micropillar arrays but simultaneously reduces the adhesion area. Enhanced cellular activities on nanostructured surfaces have been documented.^[^ ^223^ ^]^ Viela et al. studied neural stem cell morphology, proliferation rate, and cell mobility on micropillars with 500 nm and 2 μm diameters, reporting that cells cultured on smaller pillars were more spread, less circular, and exhibited numerous filopodia.^[^ ^193^ ^]^ Similarly, Holland et al. investigated murine embryonic fibroblasts and human mesenchymal stem cells on pillars with 800 nm and 1.8 μm diameters.^[^ ^29^ ^]^ They maintained equal effective stiffness across different pillar sizes by adjusting pillar height, noting that cells were less spread on pillars with smaller diameter. Given the variability in cell types and pillar configurations, generalizing these results is challenging.

Cheng et al. isolated the effects of nanotopography using four micropillar arrays with equal stiffness, each covered with different materials: plain PDMS, nanopillars, silicon oxide, and titanium oxide.^[^ ^136^ ^]^ MC3T3‐E1 osteoblast cells cultured on these arrays showed increased cell elongation, migration rates, and higher CTFs on nanopillar‐covered surfaces compared to flat PDMS and oxide‐covered ones. They also explored how substrate surface energy influences cellular responses.

Endocytosis is the process by which cells internalize external molecules by engulfing them. Benjamin et al. studied cell culturing on nano‐ and micropillars, revealing that microtopography can modify the endocytosis process.^[^ ^49^ ^]^ Their findings varied across different cell types, including human mesenchymal stem cells, MCF7 cancer cells, and COS7 monkey kidney fibroblasts.

Cell contact guidance refers to the changes in the orientation of cells and subcellular structures, finally leading to directional migration, influenced by the anisotropic geometrical features of their environment.^[^ ^224^ ^]^ While continuous anisotropic features such as microgrooves are well explored in contact guidance studies,^[^ ^225^ ^]^ anisotropic micropillar arrays and micropillars combined with directional cues also provide a competent platform for studying contact guidance and associated forces.^[^ ^14^, ^226^, ^227^, ^228^ ^]^ Park et al. used micropillar arrays with varying row and column spacing to study neurite guidance, observing clear unidirectional neurite orientation along tighter‐spaced pillars and significant growth distance increases with anisotropy, see Figure 11c.^[^ ^229^ ^]^ Leclech et al. highlighted that not only the arrangement but also the microtopography of pillars affects contact guidance.^[^ ^230^ ^]^ Their study on interneurons on arrays with square and round pillar topologies observed more oriented migration patterns on square pillars, see Figure 11d. Later, the team further discussed contact guidance on microfabricated structures in their review.^[^ ^231^ ^]^

### 5.3 Micropillars as a 3D Confinement

The ECM consists of various types of fibers that impose 3D geometric constraints on cells, with the mechanical properties of the ECM largely dependent on these fibrous structures. Various microfluidic techniques for 3D cell culture are well documented in the literature, including the use of gel substrates,^[^ ^232^ ^]^ microwells,^[^ ^233^, ^234^ ^]^ and porous scaffolds.^[^ ^235^ ^]^ By coating the lateral surfaces of micropillars with ECM proteins, researchers can direct cells into the interstitial spaces between micropillars.^[^ ^126^ ^]^ This setup serves as a spatially constrained 3D extracellular environment for research purposes.^[^ ^236^, ^237^ ^]^

Lisann et al. constructed a microcage‐like structure using micropillars to investigate 3D epithelial cell growth and homeostasis.^[^ ^140^ ^]^ They encapsulated the cell culture area with a PDMS layer atop the pillar arrangement, observing unique cell morphologies and higher contraction forces than traditional 2D cell cultures. Devine et al. employed hydrogel pillar arrays varying in pillar density and elasticity as a 3D cell culture environment.^[^ ^238^ ^]^ Here, pillar density dictated the spatial confinement, while the pillars’ elasticity adjusted the ECM's stiffness experienced by cells, see Figure 11e. Their research evaluated cell volume, traction force, and strain energy across different configurations, see Figure 11f.

Alpan et al. created an anisotropic 3D environment by confining cells within the gaps of elliptically shaped micropillars, see Figure 11g.^[^ ^239^ ^]^ They coated the pillar tops with pluronic and the side surfaces with FN to restrict cell movement within the array. Like in 2D cultures on anisotropic micropillars, cells exhibited clear elongation and growth along the stiffer direction but with significantly greater elongation lengths in this 3D setup than their 2D counterparts.

In addition to the use of micropillars as a simple cell culture environment, studies have used micropillars in organ‐on‐chip applications to imitate specific biological environments.^[^ ^240^ ^]^ In particular, micropillars show geometrical similarities with intestine villi, providing means to imitate similar artificial environments for studies of intestinal functions such as nutrition/drug absorption and barrier functions.^[^ ^241^ ^]^ Villi are fingerlike protrusions with diameters ranging from 100 to 200 μm and heights from 200 to 1000 μm.^[^ ^242^ ^]^ Researchers have utilized replica‐molded micropillars as a 3D scaffold for intestinal epithelial cell culture, where the cells form a single epithelial layer on the micropillars, accurately replicating the structure of villi.^[^ ^47^ ^]^ Caco‐2 (human colorectal adenocarcinoma) epithelial cell cultures on micropillars have demonstrated significantly higher resistance to bacterial infection compared to 2D intestine models, largely due to a substantial upregulation of mucin 17 expression.^[^ ^243^ ^]^ Additionally, cells cultured in 3D on micropillars exhibit transepithelial electrical resistance values that more closely resemble in vivo conditions compared to their 2D counterparts. Recent studies have utilized 3D‐printing and photopolymerization techniques to fabricate villi structures with more complex features, such as dual material pillars.^[^ ^242^, ^244^ ^]^ Kim et al. fabricated intestinal villi structures using hydrogel laden with endothelial cells to better mimic natural physiological conditions.^[^ ^245^, ^246^ ^]^ They report improved barrier functions of epithelial cells cultured on cell‐laden base structures compared to hydrogel‐only pillars. Macedo et al. used a similar platform to study the absorption of various drugs.^[^ ^73^ ^]^ They showed that this model can imitate transepithelial electrical resistance values and drug permeability comparable to in vivo conditions.

Gut microbiota refers to the diverse community of microorganisms that live in the gastrointestinal tract.^[^ ^247^ ^]^ These microbes play a crucial role in the host's immune response, digestion, and metabolism, and dysbiosis is associated with various diseases, including gastrointestinal and metabolic disorders, cardiovascular diseases, hypertension, depression, and even cancers.^[^ ^248^, ^249^ ^]^ Artificial intestine models consisting of micropillars are used in a number of studies to mimic the natural microenvironment of the small intestine.^[^ ^250^ ^]^ Castello et al. experimented with a similar platform to evaluate the adhesion and invasion of pathogens and probiotic therapies.^[^ ^251^ ^]^ They show that in 3D scaffold, *Lactobacillus* is more effective at displacing pathogens, while *Escherichia coli* Nissle prevents pathogen adhesion. Kim et al. developed an advanced gut‐on‐a‐chip microdevice incorporating micropillars and cyclic strain on the substrate, achieving a more accurate mimicry of in vivo conditions.^[^ ^252^ ^]^ They demonstrate successful use of probiotic and antibiotic therapies against pathogenic bacteria and notably, they were able to recreate intestine inflammations within the device.

### 5.4 Subcellular Level Response to Topology

While most of the studies have focused on the overall behavior of cells on micropillars, less attention has been given to subcellular level changes. Being the most critical organelle in the eukaryotic cells, nuclei play a major role in determining cellular functions.^[^ ^253^ ^]^ Emerging research suggests that the physical deformation of the nucleus can modulate a number of cellular functions.^[^ ^254^, ^255^, ^256^ ^]^ By controlling micropillar parameters, where cells are cultured on, nucleus can be driven in between micropillars deforming its original shape.^[^ ^257^, ^258^ ^]^ This technique shows great potential in inducing controlled large nuclei distortions compared to traditionally employed methods such as micropipette aspiration^[^ ^259^ ^]^ and force application with AFM^[^ ^260^ ^]^ for study of nuclear mechanobiology.^[^ ^261^, ^262^ ^]^

Pan et al. fabricated a series of micropillar arrays with different heights and pillar arrangements to study nuclei deformation of bone marrow stromal cells.^[^ ^258^ ^]^ According to their results, cells on the tallest micropillar array, which provided enough depth to fully submerge the cells, exhibited the most significant nuclear deformation within their study range, and they explored the ability of specially arranged micropillars to control the shape of cell nuclei, see Figure 11h. Additionally, they showed that gravity does not affect nuclei deformation by culturing cells on inverted micropillar arrays. In their experiments, despite severe nuclei deformation, bone marrow stromal cells were able to differentiate and proliferate. Moreover, another similar study revealed that nuclear deformations are not influenced by the micropillar arrays’ surface chemistry or material properties but are instead dependent on the substrate topography and cytoskeletal organization of the cells.^[^ ^263^ ^]^ Tusamda et al. proposed a mechanical model for nuclear deformation on micropillar substrates, showing that the deformation is primarily caused by actomyosin pulling forces generated between focal adhesions on the sides of the pillars and the linker of nucleoskeleton and cytoskeleton complexes.^[^ ^264^ ^]^ However, the mechanism of nuclear deformation remains an active area of research.

Mesenchymal stem cells, which are highly mechanosensitive, have been found to be particularly sensitive to nuclear deformation induced by micropillar substrates, influencing their determination of cell fate.^[^ ^265^ ^]^ Liu et al. observed mesenchymal stem cell differentiation on different height micropillar arrays, which induce different amounts of nuclei deformation.^[^ ^219^ ^]^ They found that osteogenic differentiation was enhanced with the increasing nuclei deformation. Later, the same researchers studied the nuclei deformation on micropillars in a time‐dependent manner and reported two stages of nuclei deformation. In the first stage, nuclei rapidly deform from their initial shape, and in the second stage, deformation is partially recovered slowly. They repeated the experiment with mesenchymal stem cells and osteoblasts, where they reported more prominent two‐stage deformation on stem cells.^[^ ^266^ ^]^

Generally, cancer cells show increased nuclear deformability, which plays an important role in cancer metastasis by improving their ability to pass through narrow gaps.^[^ ^267^ ^]^ Comparison of human osteoblast precursor cells and SaOs‐2 cancer cells on micropillar substrates have shown that after the initial nuclear deformation, healthy cells tend to heal the deformation, while cancer cells adapt to space between pillars by increasing the nuclear deformation.^[^ ^268^ ^]^ Nagayama et al. compared the relationship between nuclear deformation and the proliferation of healthy vascular smooth muscle cells and HeLa cancer cells cultured on micropillars.^[^ ^269^ ^]^ Both types of cells spread normally on the micropillars with significant nuclear deformation, but they report that proliferations of smooth muscle cells were significantly inhabited by the nuclear deformation compared to HeLa cells, see Figure 11i. They suggest this difference could be caused by the larger deformations of thicker nuclear lamina in vascular smooth muscle cells. In a similar study, Liu et al compared the proliferation of HeLa and HepG2 cancer cells with MC3T3‐E1 and NIH3T3 cells cultured on micropillar arrays.^[^ ^48^ ^]^ All the cell types exhibited severe nuclear deformation. However, HeLa and MC3T3‐E1 cells showed a decrease in nuclear size and proliferation, while the other two cell types did not display this behavior. These results highlight the high sensitivity of cell type to nuclear‐deformation‐induced functional changes. Ermis et al. carried out a comprehensive study on the shape of the deformed nuclei on micropillar substrates, depending on the cell type.^[^ ^270^ ^]^ They suggest that nuclear deformation has the potential to be used as a parameter for identifying cancer cells. Antmen et al. studied the correlation between malignancies of cancer cells with their nuclear deformability and rate of deformation. They tested MCF10A non‐cancerous cells, and MCF7 and MDAMB231 cancer cells.^[^ ^271^ ^]^ They reported nuclear circularity values of 0.77, 047, and 0.37, respectively, which correctly represented the malignancy levels of each cell type. They also experiment with the effect of disrupting actin filaments using drug treatments, significantly decreasing the nuclear deformation in cancer cells but not benign ones.^[^ ^272^ ^]^

## 6 Non‐Mechanobiology Applications

### 6.1 Micropillar as Electrodes

The morphology of micropillar arrays provides an intrinsic advantage due to their high effective surface area, making them frequently used as electrodes in biosensing applications.^[^ ^273^ ^]^ To maximize the electroactive surface area, smaller pillar diameters and gaps are favored. However, excessively close pillar gaps could diminish electrode performance due to the overlap of diffusion layers. As such, nanoscale pillars could show lower performance than predicted for the electrochemically active surface area due to the limited diffusivity of electroactive species compared to planar surfaces.^[^ ^274^ ^]^ This has been supported by a theoretical study demonstrating the enhanced signal through the design of a micropillar array to maximize the availability of electroactive species to the nonplanar diffusion domain.^[^ ^275^ ^]^ Also, special considerations must be addressed where pillar–electrolyte interactions occur, as micropillars are susceptible to collapse under surface tension^[^ ^276^ ^]^ and fluids may not reach pillar side and bottom surfaces due to surface tension (Cassie−Baxter state).^[^ ^277^ ^]^

The sensing principle is typically based on electron transfer from the redox reaction of electroactive species occurring on the electrode surface.^[^ ^278^ ^]^ Chen et al. focused on micropillar‐array‐based electrode design and sensitivity optimization.^[^ ^279^ ^]^ They fabricated micropillar arrays using PDMS replica molding, and a gold layer was sputtered onto the pillars to make them conductive. The experiments were aimed at identifying sarcosine and H_2_O_2_, sarcosine as a probable biomarker for prostate cancer and H_2_O_2_ as a product of the oxidation of various biomarkers, including glucose and cholesterol. Voltammetry readings were used for measurements, revealing a fully linear correlation between current density and effective surface area of micropillar array electrodes. Electrode sensitivity was further enhanced by coating the pillar array with platinum–palladium/multi‐walled carbon nanotubes. This coating improves surface area and sensitivity by introducing nanostructures on the surfaces and the excellent conductivity of carbon nanotubes. They reported more than double the sensitivity improvement from micropillars and ≈8 times greater sensitivity from micropillars with nanocoating than planar electrodes. Movilli et al. in their experiment with gold‐coated micropillars with varying IDs, show, rather than the increase of projected area, when increasing the pillar density, the effect of pillar surface roughness caused by manufacturing processes can also further improve the electrode performance.^[^ ^280^ ^]^ They show a sevenfold improvement in surface‐based electrochemical sensitivity of DNA with a micropillar array compared to the flat surface prepared with the same gold coating procedure.

#### 6.1.1 Glucose Sensing

Fast and accurate glucose sensing is critical for effective diabetes management. Micropillars are utilized in both invasive and noninvasive glucose sensors, a topic extensively discussed in the literature.^[^ ^281^ ^]^ Most commonly, glucose sensors operate on the enzymatic glucose sensing principle, using glucose oxidase (GOx) as a catalyst for glucose oxidation. The electron transfer in this reaction helps estimate glucose concentration. Micropillars offer a larger effective area for electron transfer and additional volume for enzyme encapsulation, enhancing sensor performance.^[^ ^282^ ^]^ Recent studies have also highlighted micropillar‐based nonenzymatic glucose sensors. These sensors employ metal oxide or heavy‐metal‐coated pillars to catalyze glucose oxidation, providing an alternative to traditional enzymatic methods.^[^ ^283^, ^284^ ^]^

**Figure 12:**
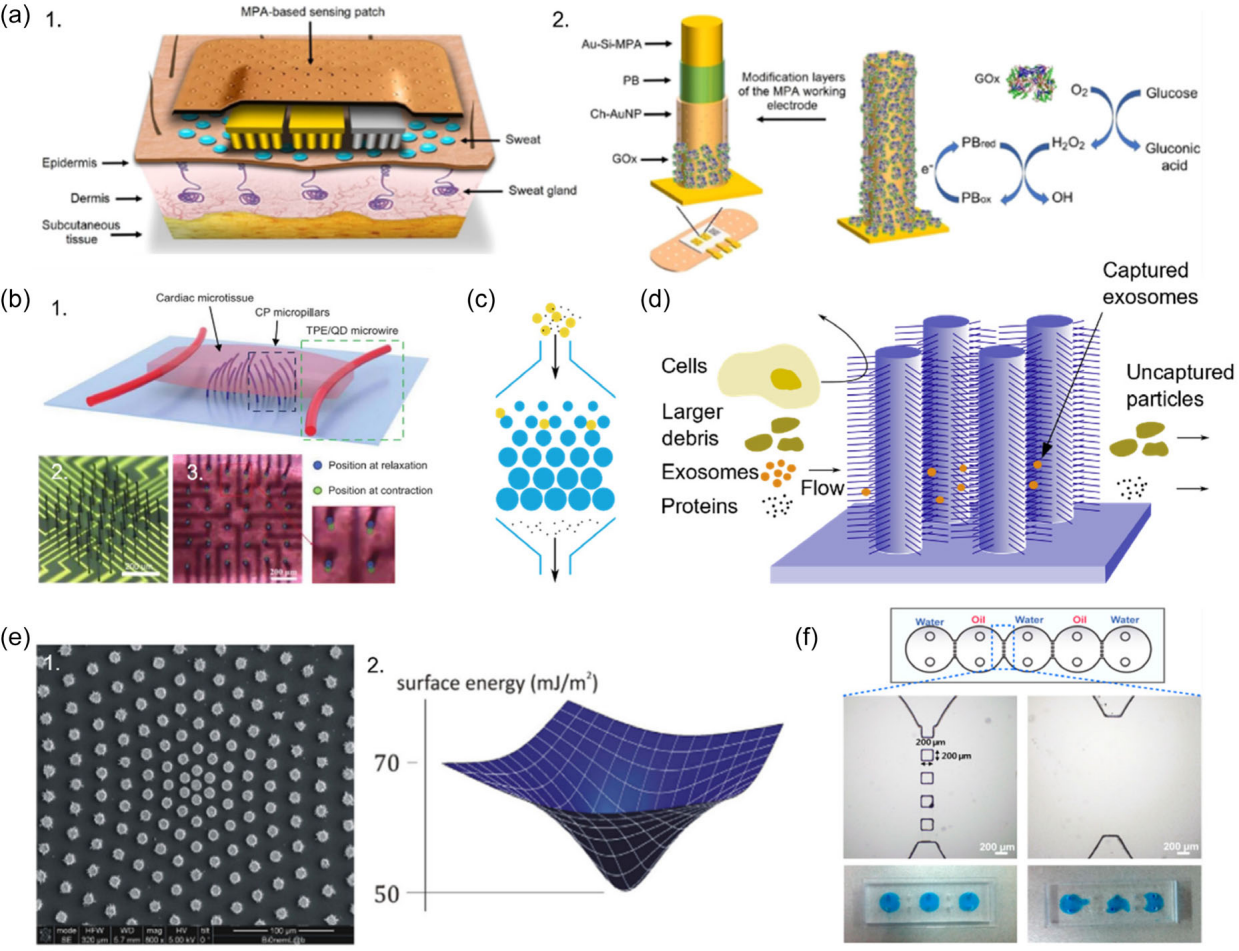
Non‐mechanobiology‐related applications of micropillars. a) Schematic diagram showing a wearable enzymatic sweat glucose sensor patch comprising arrays of counter, working, and reference electrodes. The micropillar facilitates electron exchange reactions, with Persian blue catalyzing the electroreduction of H_2_O_2_. Direct reduction of H_2_O_2_ can be also used for detection. Reproduced with permission.^[^ ^285^ ^]^ Copyright 2022. American Chemical Society. b) Cardiac tissue electrophysiological recording with flexible micropillar electrodes. Sensor measure tissue contraction forces using a Qdot‐embedded thermoplastic elastomer microwire. The array features individual electrical connections for each flexible micropillar, allowing localized electrophysiological recording at each location while maintaining minimum mechanical interference with cells. Reproduced under terms of CC BY‐NC 4.0.^[^ ^292^ ^]^ Copyright 2023, The Authors. Published by IOP Publishing. c) Micropillar‐based filtration. Interpillar distance adjustment controls particle size transfer between stages, with lateral extraction techniques applicable at each stage. d) Capturing exosomes using ciliated micropillars. Reproduced with permission.^[^ ^59^ ^]^ Copyright 2013, Royal Society of Chemistry. e) Controlling surface energy using interpillar distance. The first figure shows the pillar arrangement, and the second figure shows the surface energy variation over the array. This apparatus is used to transport specific molecules within a fluid droplet. Reproduced with permission.^[^ ^347^ ^]^ Copyright 2013, Elsevier. f) Fluid interface stabilization using micropillars. Two figures show fluid interfaces with and without micropillar arrays. This array facilitates particle passaging through the fluid interface while maintaining the separation. Reproduced with permission.^[^ ^349^ ^]^ Copyright 2015, Elsevier.

Noninvasive sensors, which utilize sweat, saliva, urine, tears, and breath for glucose testing, are gaining popularity due to their less painful testing procedures and the convenience of continuous monitoring. However, these fluids contain significantly lower glucose concentrations than blood, necessitating the development of more sensitive sensors for noninvasive applications.^[^ ^281^ ^]^ In a recent study, Dervisevic et al. developed an enzymatic sweat glucose sensor consisting of micropillar arrays for continuous glucose measurement.^[^ ^285^ ^]^ They functionalized silicon micropillars by depositing a Prussian blue layer followed by a chitosan–gold nanoparticle composite and GOx. The glucose concentration was measured by the reduction of H_2_O_2_ produced from glucose oxidation, see **Figure** **12**a. The sensor demonstrated excellent repeatability, retaining about 98% of its initial response after seven uses. Unlike planar electrodes, in a micropillar array, GOx is primarily immobilized on the sidewalls and bottom of the pillars, protecting the enzyme layer from friction damage that could occur with continuous wear of the sensor.

#### 6.1.2 Electrophysiological Recording

Electrophysiological recording is crucial for studying electrogenic cells in both neurobiology and cardiology. Micro/nanopillars are frequently utilized as electrodes for both extracellular and intracellular electrophysiological recordings.^[^ ^286^ ^]^ Various pillar‐like geometries have been explored in experiments to optimize the electrode‐cell connection. These geometries allow for either penetration or tight contact with the cell membrane making them superior compared to planer electrodes.^[^ ^287^ ^]^ Typically, pillars in these arrays are individually addressable, with necessary electronics integrated within the system.^[^ ^288^ ^]^

Heart on a chip is a relatively new tool that emerged in cardiac tissue engineering, which enables in vitro testing of cardiac drugs and disease modeling. Micropillars are popular with heart‐on‐chip applications as they allow cardiomyocytes to grow into 3D geometries and be subjected to mechanical force measurement, as discussed in previous sections, while simultaneous electrophysiological recording.^[^ ^289^, ^290^ ^]^ The conductive pillars are usually composed of Si, Pt, Au, and Ir, which possess Young's modulus in the gigapascal range. When cells penetrate or engulf these pillars, the pillars’ stiffness influences the cell's mechanical activities, especially with cardiomyocytes. As a solution, researchers have developed micropillar electrode arrays using conductive hydrogel tailored to match the mechanical stiffness of the electrodes with that of the cells.^[^ ^291^ ^]^ These arrays had shown robust signal strength and a higher signal‐to‐noise ratio than conventional electrodes. Wu et al. developed a platform featuring a flexible micropillar array capable of simultaneously recording cardiac tissue's electrophysiological signals and contraction forces while suspended in a 3D morphology.^[^ ^292^ ^]^ The cardiac tissue is supported using two nanocomposite microwires embedded with Qdots to measure contraction forces. The micropillars are individually addressable and coated with a soft conductive polymer, see Figure 12b. This platform facilitates a broad range of multiparametric recordings of cardiomyocyte activity, proving invaluable for biological studies such as cardiac drug screening.

The use of micropillar arrays in neuroscience studies for electrophysiological recording is increasingly evident due to their ability to provide less noise through tight contact with cells, high spatial resolution, and the ability to control neuron growth geometry.^[^ ^293^ ^]^ However, the use of engineered microelectrode arrays, such as Utah and Michigan arrays for neuron interfacing, are long‐established techniques with similarities to micropillars.^[^ ^294^ ^]^ Compared to 2D electrodes, Spanu et al. showed that micropillar electrodes exhibit higher signal amplitudes and powers with 4‐4‐Aminopyridine‐induced epileptiform activities on brain slices.^[^ ^295^ ^]^ Similar to experiments with cardiomyocytes, flexible micropillars also show promising advancements in neural electrophysiological recordings through enhanced signal‐to‐noise ratios.^[^ ^296^, ^297^ ^]^ These techniques can greatly benefit brain organoids on‐chip studies, which aim to mimic the structure and functionality of brain tissues on chips.^[^ ^298^ ^]^ Going beyond in vitro electrophysiological recording, micropillar electrodes are also being experimented as neural probes for brain interfaces based on their ability to penetrate cellular assemblies.^[^ ^299^ ^]^ These micropillar electrode arrays show great potential in neuroprosthetics, which aims to facilitate direct communication between the brain and electronics.^[^ ^300^ ^]^ Recent advancements in similar neural interfaces, their applications, and design strategies, including improvements in biocompatibility and flexibility, have been reviewed elsewhere.^[^ ^294^, ^297^, ^301^ ^]^

Cellular stimulation refers to the application of external mechanical, electrical, chemical, or optogenetic stimuli to cells to trigger a physiological response.^[^ ^302^, ^303^, ^304^ ^]^ With tunable resolution, flexibility, and the ability to penetrate tissues, micropillar arrays offer a versatile platform for cellular stimulation across various applications. Micropillars have proven highly effective in delivering mechanical stimuli to cells, due to their unique geometrical properties. While magnetically actuated micropillars are commonly used in mechanostimulation studies, optomechanical stimulation has also been explored.^[^ ^305^, ^306^ ^]^ Zhu et al. developed a micropillar‐like bio‐dart to induce transient pressures on neuron cells by utilizing optical scattering forces generated by a laser to propel the dart.^[^ ^304^ ^]^ This technique enabled subcellular resolution and tunable transient pressures on the cell membrane through adjustments in laser beam parameters. Their study demonstrated the activation of Piezo1 ion channels, which are highly mechanosensitive, via bio‐dart stimulation. Furthermore, they successfully applied this technique for in vivo neural stimulation in zebrafish larvae.

In addition, conductive micropillars provide a precise and high‐resolution method for delivering electrical stimulation to cells, especially nerve cells.^[^ ^307^ ^]^ Electrical stimulation of nerve cells is a widely used technique in neuroprosthetics. Wang et al. fabricated a carbon nanotube electrode array for neural stimulations, which shows several advantages over conventional metal electrodes owing to its high charge injection limit, strength, and flexibility.^[^ ^308^ ^]^ Tomaskovic‐Crook et al. compared the neural stem cell differentiation and morphology on conventional plate‐based culture and conductive micropillar arrays with electrical stimulations.^[^ ^309^ ^]^ Stimulated cells showed more organized and densely packed neural construction with long‐distance axonal projections and synaptic connections. Cardiac tissues are another type of tissue that is often studied under electrical stimulation.^[^ ^310^ ^]^ Zhang et al. developed a flexible micropillar platform capable of supporting the growth of 3D cardiac tissues while integrating electrophysiological recording and electrical stimulation capabilities.^[^ ^289^ ^]^ Electrical stimulation can enhance tissue function by altering structural dynamics and promoting contractile development.

### 6.2 Surface‐Based Biosensing

Beyond electron exchange identification, micropillar arrays have also been utilized to enhance the efficiency of surface‐chemistry‐based biosensing. Micropillars provide increased area for molecular or cell binding, and their morphological features can enhance and manipulate the interactions between the surfaces and targets. Ahmed et al. developed such a platform for immunocapturing circulating tumor cells (CTC) in blood.^[^ ^311^ ^]^ They implemented antibody‐coated triangular micropillars as a deterministic lateral displacement (DLD) array to use hydrodynamic effects to selectively enhance the interaction of CTC with micropillars. They reported 92% efficiency and 82% purity in capturing 1000 SW480 cells mixed with 1 mL of healthy blood. Tumor‐derived extracellular vesicles can also be used as a biomarker for early tumor detection. Kamyabi et al. developed a similar platform consisting of a zigzag micropillar array coated with antibodies against extracellular vesicle surface proteins or a cancer‐specific antigen to capture extracellular vesicles from plasma.^[^ ^312^ ^]^ They report comparable results and significantly lower processing times compared to ultracentrifugation, which is the gold standard for extracellular vesicle isolations, showing the potential of this system as a point‐of‐care testing device. In another experiment, Malara et al. manipulated the surface energy of a super hydrophobic micropillar array to enhance the detection of reactive species produced by tumor metabolism in the cultivation medium.^[^ ^313^ ^]^ Marangoni flows developed within the droplet by varying surface energy cause spatial separation of biological species. This technique is similar to the method shown in Figure 12e.

Clustered, regularly interspaced short palindromic repeats (CRISPR)‐technology‐based pathogen detection systems are currently becoming popular due to their excellent sensitivity and specificity. The use of micropillar arrays for enhancing CRISPR techniques is still limited but shows great potential. Hass et al. developed a solid‐phase viral DNA detection platform utilizing CRISPR‐Cas12a.^[^ ^314^ ^]^ They immobilized the reporter probes on the surface of PDMS micropillars, which provided an enhanced surface area for binding. They report ≈40% higher probe binding capacity in micropillar‐covered channels than in flat channels. Bao et al. studied the micropillar density variation on molecular binding capacity and detection sensitivity in CRISPR‐based human papillomavirus detection.^[^ ^315^ ^]^ They utilized high aspect ratio micropillars with a 25 μm diameter and 300 μm height with varying pitch distances of 109, 146, and 197 μm. They report that the first two pitch distances show ≈5 and 3 times higher molecular binding capacity relative to the 197 μm pitch distance. For the detection, they introduced fluorescence resonance energy transfer (FRET) to the platform with Qdots as FRET donors.

Enzyme‐linked immunosorbent assay (ELISA) is an analytical technique mainly used to detect and quantify protein biomarkers. Generally, in ELISA, a capture molecule is first immobilized on a solid surface, and subsequently, target molecules are allowed to bind to it. Then, a detection antibody conjugated with an enzyme is added to bind with target molecules, and the remaining are washed away. Finally, a substrate is added to the system to produce a detectable reaction catalyzed by the enzyme bound to the target molecule through the detection antibody.^[^ ^316^ ^]^

Microfluidic platforms can improve the efficiency of ELISA procedure through greatly reduced sample volumes and efficient micromanipulation of fluids and engineered surfaces with improved surface binding.^[^ ^316^, ^317^, ^318^, ^319^ ^]^ Integration of micropillar arrays with ELISA can significantly improve the effective surface area of detection solid phase. Geissler et al. performed a series of experiments with varying pillar diameter, height, and pitch over a wide range, to evaluate the effect of micropillar geometrical parameters on solid‐phase immunoassays. Their experiment with 12 different micropillar array parameters shows an excellent linear relationship with adequate surface area and antibody detection performance within the tested range. Numthuam et al. modified the nanoscale surface properties of a PDMS micropillar array through gold‐black coating for electrochemical detection of bone alkaline phosphatase, which is a bone metabolic biomarker.^[^ ^320^ ^]^ They used p‐aminophenyl‐b‐D‐galactopyranoside as the substrate converted to electroactive p‐aminophenol by conjugated β‐galactosidase. Cyclic voltammetry readings detected the produced p‐aminophenol. They reported a 9.2 times higher detection current in gold‐black‐coated micropillar electrodes than in plain gold electrodes. Even with improved surfaces, limited mass transport of target molecules to the solid phase could result in long incubation times and low efficiency. Zhou et al. integrated an ultrasound transducer beneath the micropillar platform to generate a more chaotic flow within the device and enhance binding.^[^ ^321^ ^]^ Ultrasonic vibrations create microvortices around the micropillars, enhancing the detection limit of thrombin up to 10 times compared to the same micropillar array without an acoustic field. As a more straightforward method, Suzuki et al. tested a motorized rotating stirrer device by stacking films with micropillar arrays, which can be inserted into wells in a 96‐well plate.^[^ ^322^ ^]^ The rotation causes solutions to flow through micropillar arrays continuously, improving the chance of contact with the surface and simultaneously reducing the incubation time at each step of ELISA.

### 6.3 Micropillar Geometry‐Based Applications

Micropillar fabrication techniques precisely control pillar parameters and arrangement, making micropillar arrays useful as passive filtering or sorting mechanisms in lab‐on‐chip (LOC) applications and often integrated upstream of the sensing region.^[^ ^323^ ^]^ The gaps between micropillars can be utilized for physical filtration, and arrays with varying gaps can be employed to separate multiple types of particles from a mixture, see Figure 12c. This technique is notably applied to isolate CTCs from blood.^[^ ^324^, ^325^ ^]^ Extracellular vesicle isolation is a relatively new field that can significantly benefit from microfluidics.^[^ ^326^ ^]^ Wang et al. used a micropillar array with nanopillar‐covered surfaces to isolate exosomes, which range from 10 to 100 nm in diameter.^[^ ^59^ ^]^ The inter‐micropillar gap was set to prevent larger cells from entering the filtering area, while the nanopillars only captured exosomes by letting other particles pass through the filtering region. Subsequently, exosomes are released by dissolving the nanopillars, see Figure 12d.

#### 6.3.1 DLD

Introduced by Huang et al. in 2004, DLD is a continuous particle separation/sorting technique that operates through micropillar array‐induced hydrodynamic forces,^[^ ^327^ ^]^ and is widely used in biosensing applications. DLD can be used to facilitate the separation of red blood cells, white blood cells, and CTCs from blood and several other bioparticles.^[^ ^328^, ^329^, ^330^ ^]^ Numerous comprehensive reviews are available on the theoretical aspects, design considerations, and advancements of DLD‐based particle separation.^[^ ^331^, ^332^, ^333^ ^]^

#### 6.3.2 Liquid Chromatography

Liquid chromatography is frequently used in biology to separate proteins, nucleic acids, and other molecular mixtures. Traditional liquid chromatography uses a packed bed of particles as the stationary phase, where the interaction between the mobile phase and the stationary phase causes differential movement of components, facilitating separation.^[^ ^334^ ^]^ Particle size and geometrical packing of the packed bed profoundly affect the performance of a setup.^[^ ^335^ ^]^ Utilizing regularly ordered micropillar array columns as the stationary phase is an innovative approach that offers higher efficiency by reducing band broadening, improving separation resolution, and reducing back pressure.^[^ ^336^ ^]^ Compared to particle‐packed beds, micropillar array devices do not require bed retainers as pillars are stably attached to the underlying substrate, and these platforms demonstrate outstanding repeatability. As micropillars are fabricated with a defined mask, the reproducibility of the device is also excellent.^[^ ^337^ ^]^ The Thermo ScientificμPAC Neo high‐performance liquid chromatography (HPLC) Columns, a commercially available micropillar‐enhanced liquid chromatography system originally developed by PharmaFluidics, is widely used in proteomics studies.^[^ ^338^, ^339^ ^]^

#### 6.3.3 Wettability‐Based Droplet and Biomolecule Manipulation

Micropillar arrays often exhibit unique hydrophobic properties that can be manipulated via pillar arrangement geometry^[^ ^340^, ^341^ ^]^ and lattice parameters.^[^ ^342^ ^]^ These properties are experimented with in defined droplet transportation on surfaces owing to the broad spectrum of applications in LOCs and biosensors.^[^ ^343^, ^344^ ^]^ In these experiments’ liquid droplets are transported on the surfaces by alternating wettability through mechanical stretching or magnetically actuating micropillars in a defined pattern.^[^ ^345^ ^]^

Beyond droplet transportation, Angelis et al. explored micropillar arrays in biomolecule manipulation within a droplet to direct targeted molecules to specific sensing locations, which is challenging in highly diluted solutions.^[^ ^346^ ^]^ A wettability gradient through the pillar arrangement can condense targeted molecules to specific predefined points, see Figure 12e.^[^ ^347^ ^]^ A further developed variant of this technique has been used to separate and identify multiple biomolecules in a single droplet.^[^ ^348^ ^]^

Kim et al. utilized micropillar‐geometry‐based surface properties in an LOC enzyme‐linked immunosorbent assay to enhance the identification of amyloid *β*, a biomarker for Alzheimer's disease.^[^ ^349^ ^]^ On the microchip, micropillars stabilize the interface between consecutive assay chambers containing aqueous solutions and oil, allowing magnetic droplets to transport from one medium to another without mixing the two liquids, see Figure 12f.

## 7 Conclusion and Future Work

This review explored the utilization of micro/nanopillars in biological sensing with a primary focus on cell mechanobiology. We discussed the design, fabrication, and measurement techniques of micropillars, highlighting their versatility and precise capabilities in studying cellular biomechanics. Since their introduction in the early 2000s, micropillars have been adopted in cell biology as a robust alternative for measuring CTFs and modulating ECM properties.

Fundamental biological processes such as cell migration, proliferation, and morphogenesis are intricately linked to mechanical forces, underscoring the importance of accurate force measurement in understanding cellular behavior. Innovations in micropillar design, such as local or whole‐cell mechano‐stimulation capabilities, have further enriched our understanding of cell mechanosensing mechanisms. Additionally, the ability of micropillars to mimic anisotropic mechanical properties and 3D ECM properties provides a unique platform to study cell mechanobiology aspects, often unmet by conventional methods. Moreover, micropillar‐based techniques extend beyond mechanobiology, including enhancements in electrode performance, surface‐chemistry‐based biosensing, filtration, and fluid manipulation applications. The versatility of micropillar arrays, especially when combined with other microfluidic techniques like nanoscale surface modifications, integrated electronics, optical properties, and active fluidic control, significantly broadens their potential applications across diverse scientific fields.

Despite these advancements, certain limitations persist. The fabrication of pillars with varying heights, particularly those with an actual gradient, remains a significant challenge due to the complexities of multistep lithography. Furthermore, micropillar deflection measurements rely heavily on visual techniques, where challenges such as resolution limits in visualization and accurate displacement measurement prevail. While newer visualization methods like quantum dots embedded in pillars have evolved, the necessity for line‐of‐sight observation remains a significant barrier, especially for in vivo applications and the development of point‐of‐care testing platforms. Looking forward, the ideal micropillar system for CTF measurement might incorporate remote force measurement techniques for each pillar.

Although micropillar platforms are prevalent in research settings, their commercialization is still limited. Micropillar‐based CTF sensing, particularly as a cancer biomarker within lab‐on‐a‐chip devices for cancer detection, shows potential but faces practical challenges, such as the need for sophisticated imaging and displacement tracking systems that complicate point‐of‐care applications. Further, micropillar‐based tuning of substrate properties has demonstrated effectiveness in enhancing human mesenchymal stem cell differentiation into osteogenic cells, which is beneficial for bone healing. While micro/nanostructures on bone implants are already being experimented,^[^ ^350^ ^]^ the development and commercialization of implants with precisely tuned micropillars that optimize bone healing remain areas ripe for advancement.

Micropillar‐enhanced, noninvasive sweat glucose sensors represent a significant advancement in continuous glucose monitoring. Given the prominence of glucose sensors as the largest market segment within biosensors, a sector experiencing rapid growth, these micropillar‐based sensors are poised for substantial commercial success as wearable glucose monitors. Their ability to perform accurate and continuous glucose level assessments noninvasively aligns with current health monitoring trends, emphasizing convenience and patient comfort. Micropillar‐based enhancements in CRISPR techniques and ELISA are promising for faster and more effective point‐of‐care disease identification. Additionally, micropillar‐based fluid manipulation systems, such as DLD, can further improve biosensor specificity through continuous upstream separation of bioparticles, facilitating a range of analyses from clinical diagnostics to complex biochemical research. Liquid chromatography, a staple in molecular‐level mixture separation, has seen significant enhancements by integrating micropillar arrays. These enhancements have been successfully commercialized over the past few decades, with systems like the Thermo Scientific μPAC Neo HPLC leading the way. Predominantly utilized in proteomics research, these micropillar‐enhanced systems offer higher efficiency, better separation resolution, and lower back pressures than traditional liquid chromatography systems.

Despite these advancements, the utilization of micropillar technology in point‐of‐care testing and wearable sensing devices remains underdeveloped. While micropillar‐based systems have proven their utility in controlled research environments, translating these benefits to practical, everyday applications presents the next frontier. Enhancing micropillar‐based devices’ robustness, user‐friendliness, and integration capabilities will be crucial for their adoption in point‐of‐care settings and personal health monitoring. As the demand for more sophisticated biosensing capabilities grows, particularly in personalized medicine and continuous health monitoring, the potential for micropillar technologies to transform these areas is immense. Future research and development will likely focus on overcoming the current limitations and expanding the practical applications of this promising technology.

## Conflict of Interest

The authors declare no conflict of interest.

## Author Contributions

**Prabuddha De Saram**: data curation (lead); formal analysis (lead); investigation (lead); methodology (equal); software (lead); visualization (lead); writing—original draft (lead). **Nam‐Trung Nguyen**: project administration (lead); supervision (equal); validation (equal); writing—review & editing (equal). **Sina Jamali**: Formal analysis (lead); validation (equal); writing—review & editing (equal). **Navid Kashaninejad**: formal analysis (lead); funding acquisition (lead); project administration (equal); resources (lead); supervision (lead); writing—original draft (supporting); writing—review & editing (equal).
